# TLR4 and NKT Cell Synergy in Immunotherapy against Visceral Leishmaniasis

**DOI:** 10.1371/journal.ppat.1002646

**Published:** 2012-04-12

**Authors:** Subir Karmakar, Siddhartha Kumar Bhaumik, Joydeep Paul, Tripti De

**Affiliations:** Division of Infectious Disease and Immunology, Indian Institute of Chemical Biology, Council of Scientific and Industrial Research, Kolkata, India; London School of Hygiene and Tropical Medicine, United Kingdom

## Abstract

NKT cells play an important role in autoimmune diseases, tumor surveillance, and infectious diseases, providing in most cases protection against infection. NKT cells are reactive to CD1d presented glycolipid antigens. They can modulate immune responses by promoting the secretion of type 1, type 2, or immune regulatory cytokines. Pathogen-derived signals to dendritic cells mediated via Toll like Receptors (TLR) can be modulated by activated invariant Natural Killer T (iNKT) cells. The terminal β-(1–4)-galactose residues of glycans can modulate host responsiveness in a T helper type-1 direction via IFN-γ and TLRs. We have attempted to develop a defined immunotherapeutic, based on the cooperative action of a TLR ligand and iNKT cell using a mouse model of visceral leishmaniasis. We evaluated the anti-*Leishmania* immune responses and the protective efficacy of the β-(1–4)-galactose terminal NKT cell ligand glycosphingophospholipid (GSPL) antigen of *L. donovani* parasites. Our results suggest that TLR4 can function as an upstream sensor for GSPL and provoke intracellular inflammatory signaling necessary for parasite killing. Treatment with GSPL was able to induce a strong effective T cell response that contributed to effective control of acute parasite burden and led to undetectable parasite persistence in the infected animals. These studies for the first time demonstrate the interactions between a TLR ligand and iNKT cell activation in visceral leishmaniasis immunotherapeutic.

## Author Summary

Kala azar (visceral leishmaniasis) is a deadly disease caused by the parasitic protozoa *Leishmania donovani*. In absence of a suitable vaccine, the incidence of leishmaniasis has increased. The World Health Organization observes that, if the disease is not treated, the fatality rate in developing countries can be as high as 100% within 2 years. Therapy of visceral leishmaniasis can be complicated by toxic side effects, drug resistance, and the need for prolonged treatment regimens. Therefore, improved therapy for leishmaniasis remains desirable. Immunotherapy to selectively induce type 1 immune responses considered essential for resistance to leishmaniasis has shown great promise. CD1d-binding glycolipids stimulate TCR signaling and activation of invariant natural killer T (iNKT) cells. Terminal β-(1–4)-galactose residues in glycoconjugates have been identified as the TLR ligand that induces IFN-γ via TLR signaling. We have used the β-(1–4)-galactose terminal glycosphingophospholipid (GSPL) antigen from *L. donovani* parasites to treat infected BALB/c mice. We report that immunotherapy with GSPL induced IFN-γ, a type 1 cytokine, through the cooperative action of TLR4 and NKT-cells that contributed to effective control of acute parasite burden in the infected animals.

## Introduction

Visceral leishmaniasis (VL) is a deadly disease caused by the parasitic protozoan *Leishmania donovani* (*LD*) in India, Bangladesh, China, Nepal and Sudan; by *L. Infantum* in N. Africa and Southern Europe, and by *L. chagasi* in Latin America. There is a regional variation in response to antileishmanial drugs and thus treatment regimens vary in different regions. Except for in Europe and antimony-unresponsive regions of India, pentavalent antimonials are still the drug of choice. In Europe liposomal amphotericin is used. Miltefosine is the first effective oral drug for VL. http://www.nature.com/nrmicro/journal/v5/n11/full/nrmicro1748.html - B119#B119Most available drugs are costly, cause severe side toxicity, require long treatment regimens and are becoming more and more ineffective, necessitating the discovery of new drugs. The problem is further magnified by the emergence of drug resistance and HIV co-infection [1], [2]. One approach that has shown promise is immunotherapy.

The disease is characterized by depressed cell-mediated immunity (CMI) and agents which directly stimulate the macrophage (Mφ) to kill intracellular amastigotes and/or induce the basic T-helper type 1 (Th1)-cell anti-leishmania immune response would provide a rationale for treatment in visceral infection [3]. Conventional CD4+ and CD8+ T cells of the immune system recognize specific peptide antigens (Ag) bound to major histocompatibility complex (MHC) class II or MHC class I molecules, respectively. In contrast Natural Killer T (NKT) cells are a unique subset of T cells that recognize glycolipid antigens presented by CD1d molecules. NKT cells have the potential to produce key type 1 and type 2 cytokines and are involved in the control of several types of immune response [4]–[6]. It has been suggested that IFN-γ production in response to glycolipid Ag stimulation is initiated after Toll-like-receptor (TLR) signaling of Ag-presenting-cells (APCs) and subsequent recruitment of NKT cells as well as other cell types [7]. The β(1–4) galactose terminal Lewis X type glycans have the ability to modulate host responses in a Th1 direction via NF-κBp65, IFN-γ and macrophage TLRs [8]. Binding of *LD* immunostimulating glycosphingophospholipid (GSPL) Ag to the *Ricinus communis* agglutinin-1 (RCA-1) [9] suggests that this glycolipid possesses terminal β1,4 linked galactosyl residues [10]. *In vitro* pulsing of *LD* infected APCs with GSPL, caused the activation of the Vα14+CD1d1-specific NKT cell hybridoma DN32.D3 [11]. GSPL also induced ROS and RNI in addition to IFN-γ and IL-12 in PBMC from normal individuals [12]. Control of *LD* infection depends on early induction of an IL-12-driven expansion of Th1 cells, production of IFN-γ [13], [14], Mφ activation and subsequent generation of reactive nitrogen and oxygen species [15]. Because GSPL can induce type 1 cytokines and nitric oxide (NO) generation in Mφs, we tested its therapeutic efficacy in the mouse model of VL. Our data demonstrated that GSPL could confer complete protection to *LD* infection by an early triggering of IL-17 and IFN-γ responses of murine NKT cells by TLR4 activated CD11c+ APCs.

## Results

### Importance of terminal β-(1–4)- galactose residues for GSPL mediated protection

To explore whether terminal β-(1–4)-galactose residues were needed for GSPL mediated protection, GSPL was treated with α or β galactosidase. The purity of these preparations was analyzed by 1) TLC mobility shift and 2) by lectin blotting with *Erythrina cristagalli* lectin (ECL), which detects terminal β1,4-galactose residues (Figure S1 A,B). Bone marrow-derived-macrophages (BMDC) adhered on cover slips were infected with *LD*. Therapeutic efficacy of galactosidase treated GSPL (100 µg/mL) was compared to untreated GSPL (100 µg/mL). Intracellular parasite number in control *LD* infected BMDC was 4310±165.44 parasites/1000 BMDC. α-galactosidase treated (100 µg/mL) and untreated GSPL (100 µg/mL) reduced the intracellular parasite number of *LD* infected BMDCs by 97.2% and 97.5% respectively (123.66±14.29 parasite/1000 BMDC and 110.33±21.59 parasite/1000 BMDC respectively). However, commercially available position specific β-(1–4)-galactosidase treated GSPL reduced the intracellular parasite number by only 2.7% (4237±79.37 parasite/1000 BMDC). These observations indicate that terminal β-(1–4) galactose residues play an important role in the therapeutic efficacy of GSPL against experimental VL. No obvious cytotoxicity was noted against BMDC at concentrations ranging from 50–400 µg GSPL/mL/10^6^ cells (Figure S1 C). A critical function of macrophages is their ability to phagocytose. The phagocytic ability of the BMDCs treated with GSPL remained unaltered (Figure S1 E).

### Therapeutic immunization with GSPL

**Figure 1:**
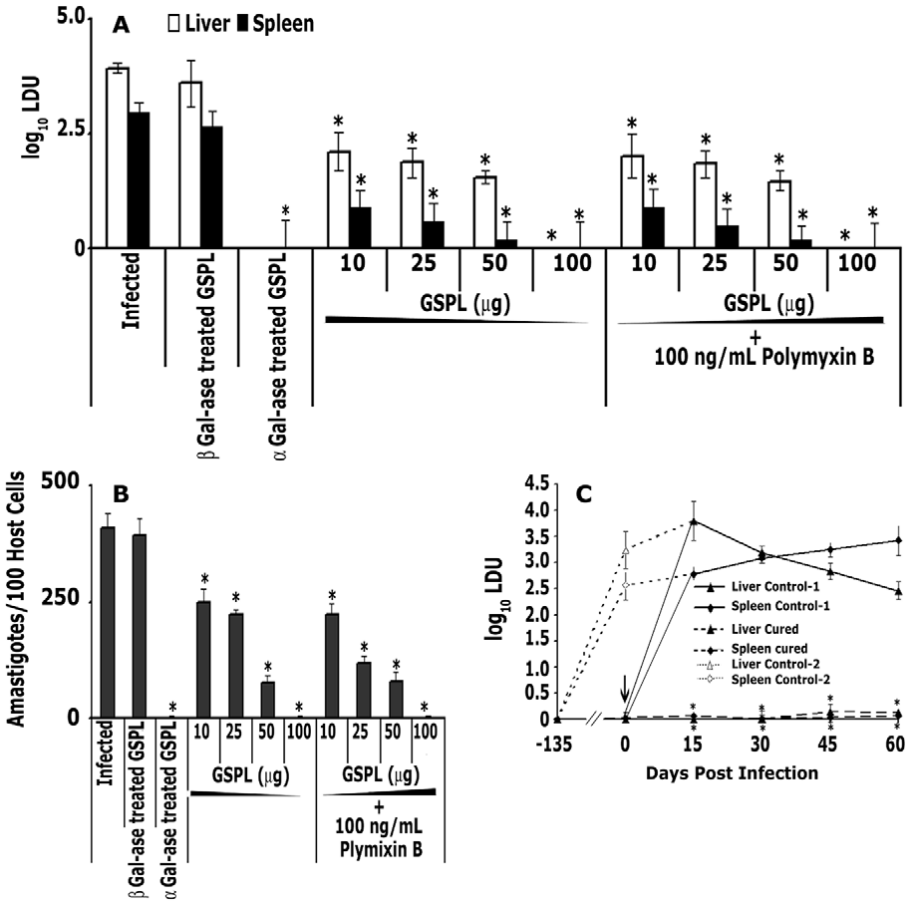
Complete cure induced in LD infected BALB/c mice following GSPL therapy. Various subcutaneous doses of GSPL were given (15 days apart) twice staring on the 60^th^ day after infection. Animals were sacrificed 15 days after therapy. Parasite loads of (A) liver, spleen, and (B) bone marrow of individual animals were determined as described in the *Materials and Methods*. (C) The course of visceral re-infection was studied by i.c. administration of 1×10^7^ *LD* promastigotes into naive, age-matched BALB/c mice and cured (100 µg/dose-GSPL treated) mice 8 wks post therapy. Naïve age-matched mice infected at the time of re-infection was kept as control-1. Mice that were infected at the initiation of the experiment and did not receive immunotherapy were kept as control-2. The progression of infection was monitored by determining the spleen and liver parasite burden by serial dilution assay, up to 60 days after re-infection. Arrow indicates the point of re-infection (day 0). Controls were infected but not vaccinated. Data represent the means ± SD of 20 animals per group, and are representative of three independent experiments. *p*<0.0001 compared with respective infected control groups at all time points; paired two-tailed Student's t-test.

GSPL binds to CD1d, restores the defective APC function in *LD*-infected cells, and stimulates robust IL-2 production in Vα14Jα18 NKT hybridoma cells [16]. The evident immunomodulatory property of GSPL paved the way for assessing its antileishmanial potency in the murine model of VL. Treatment of *LD*-infected BALB/c mice was initiated 8 wk post-infection (p.i.), time when the infection was already established. *LD* infected animals were divided into five groups of 20 animals each. Group I mice received 100 µL of vehicle only. Mice in groups II received β1,4-galactosidase treated GSPL twice at 15 days interval (100 µg/100 µL vehicle, subcutaneous [s.c.]), Group III mice received 100 µL of αgalactosidase treated GSPL twice at 15 days interval (100 µg/100 µL vehicle, s.c.), Group IV received GSPL only while group V received 100 ng/mL polymyxin/dose along with GSPL. Fifteen days post treatment, parasite burden in the liver, spleen, and bone marrow of each BALB/c mice was determined. The effect of GSPL was dose dependent, being effective at 10, 25, 50 µg/dose, and optimal at 100 µg/dose (Figure 1 A). At 100 µg/dose, there was complete absence of amastigotes in the impressions of stamp smears of transverse sections of spleens and livers of 90%, and 100% animals respectively. There was also complete absence of amastigotes in the bone marrow smears of 90% animals (Figure 1 B). Rest of the animals showed ∼99% reduction in splenic and bone marrow parasite burden. The reduction in parasite number was sustained up-to 12 months post treatment, until the end of the experiment. Up-to 12 months later, we still could not detect any liver parasite burden, while spleen and bone marrow parasite burden was still almost absent (Figure S2). At the end of treatment, the average body weight in animals treated with GSPL was equivalent to values for the control vehicle treated group (control group of mice: 25.9±1.1 g; treated group of mice: 26.1±1.2 g). There was no appreciable change in the protective efficacy of GSPL in presence of polymyxin B (100 ng/mL, Figure 1 A). This result rules out the possibility that there was any LPS (lipopolysaccharide) contamination in GSPL. The LAL endpoint assay further confirmed that the GSPL preparations did not contain endotoxin (data not shown). To further ascertain whether GSPL had conferred long-lasting immunity, cured mice were later re-infected via the intracardiac (i.c.) route, 8 wk after the last GSPL dose. Parasite burden in the re-infected animals progressed rapidly in vehicle-treated BALB/c mice, whereas GSPL-treated mice remained resistant, as observed up to 60 days (Figure 1 C). Thus, GSPL therapy might exert an acquired protective immunity against VL.

In order to confirm the association of terminal galactosylation with the protective efficacy of GSPL, *LD*-infected BALB/c mice (n=20) were treated with β1–4 galactosidase treated GSPL (100 µg/dose) or α-galactosidase treated GSPL (100 µg/dose) 8 wk p.i. As shown in Figure 1 A and 1 B, treatment of GSPL with β1–4 galactosidase resulted in only ∼4.76%, 4.8% and 3.7% reduction of hepatic, splenic and bone marrow parasite burden respectively, while α-galactosidase treated GSPL was as effective as untreated GSPL.

### GSPL mediated protection depends on TLR4

**Figure 2:**
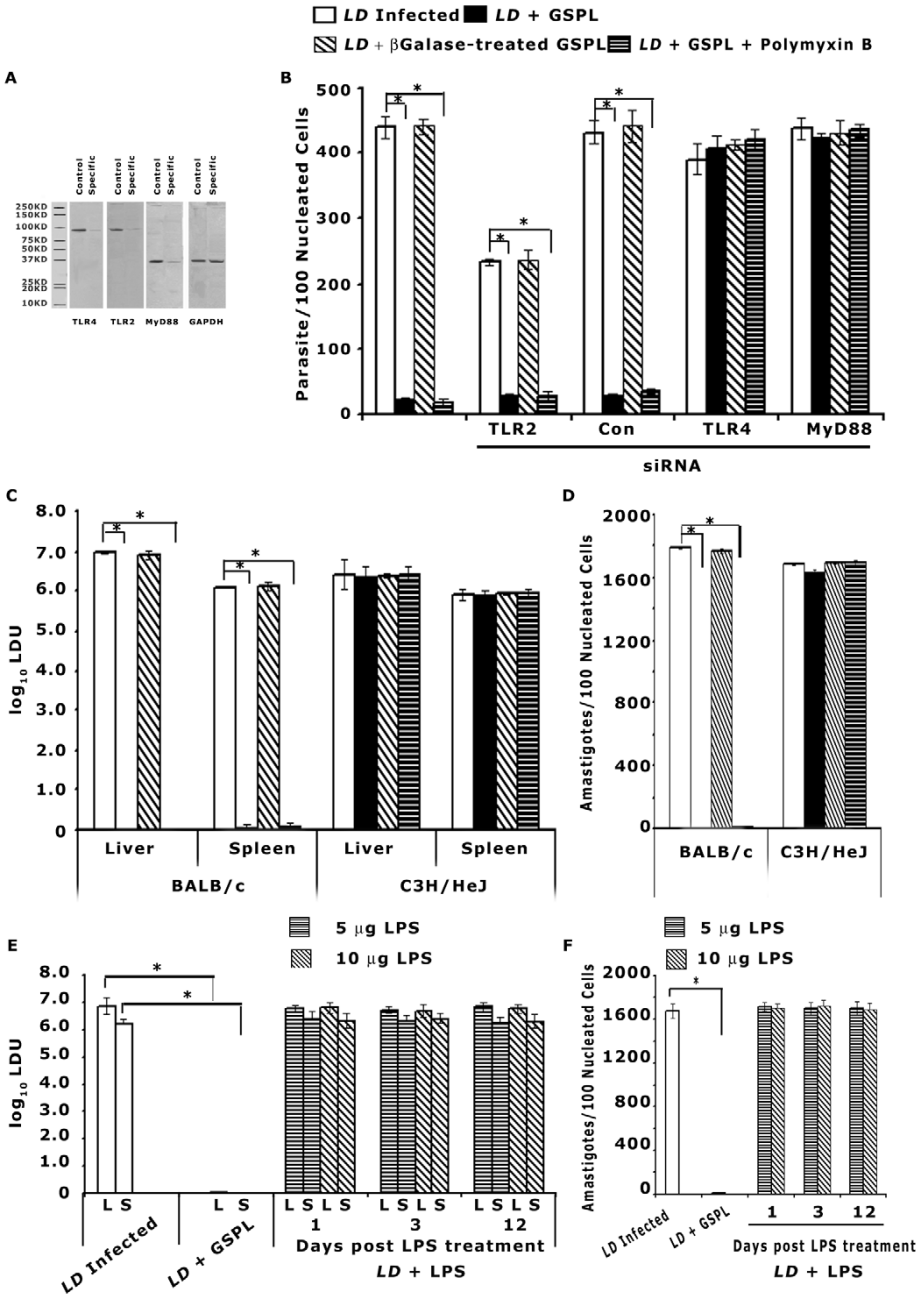
TLR4 and MyD88 are required for GSPL induced anti-leishmanial response. (A) Splenic adherent cells were transfected with siRNAs specific to TLR2, 4 or MyD88 (specific). A control group was transfected with control siRNA (control). Twenty-four hours after transfection, cells were recovered and their TLR2, 4 and MyD88 levels assessed in Western blots. GAPDH in total proteins was used as loading controls. Blots are representative of three separate experiments. (B) Splenic adherent cells transfected with TLR2, TLR4, MyD88 or control siRNA (con), were infected with *LD* (APC/parasite 1∶20) for 12 h. Non-ingested promastigotes were removed by washing, and adherent cells were cultured for another 36 h. Infected APCs were then treated with GSPL (100 µg/mL) for 24 h. Intracellular parasite number was determined by Giemsa staining. Each experiment was conducted in triplicate and repeated at least three times each and one set of representative data is shown. Error bars represent mean ± SD. * p<0.0001; paired two-tailed Student's t-test. (C,D) Antileishmanial effect of GSPL on parasite growth in TLR4 deficient C3H/HeJ mice. Sixty days *LD* infected C3H/HeJ mice were given two subcutaneous injections of GSPL (100 µg each) at 15 days apart. The parasite burdens in liver (C), spleen (C), and bone marrow (D) of individual animals were then determined at 15 days after the last treatment. The results are representative of three independent experiments and data shown are means ± SD; n=5. *p<0.0001 versus corresponding infected control; paired two-tailed Student's t-test. (E,F) *In vivo* parasite load in liver, spleen and bone marrow of *LD* infected BALB/c mice treated with LPS. Sixty days *LD* infected BALB/c mice were given three intraperitoneal injections of LPS on alternate days (5 µg/injection or 10 µg/injection). The parasite burdens in liver (E), spleen (E), and bone marrow (F) of individual animals were then determined at days 1,3 and 12 after the last treatment. The results are representative of three independent experiments and data shown are means ± SD; n=3. *p<0.0001 versus corresponding infected control; paired two-tailed Student's t-test.

It has been reported that the terminal β-(1–4)-galactose linked glycans can modulate host responses in a T-helper type 1 direction via NF-κB p65, IFNγ and macrophage TLRs [8]. As GSPL expresses β1,4-terminal galactose residues, we designed experiments to determine whether TLR expression on APCs was important for intracellular parasite killing by GSPL. Treatment of infected BMDCs with 100 µg/mL GSPL but not β-galactosidase treated GSPL resulted in a significant reduction in intracellular amastigotes (96% reduction) and this reduction was notably negated by prior treatment with TLR4/MD2 Ab (10 µg/mL), but not TLR2 Ab (10 µg/mL) (2% and 92% reduction in intracellular parasites respectively) (Figure S3 A). The viability (Figure S3 B) and phagocytic ability (Figure S3 D) of the BMDCs treated with GSPL remained unaltered. To further assess the possible role of TLR2 and 4 in GSPL-mediated anti-leishmanial effector response, siRNA mediated knock-down system was used. TLR silencing was confirmed by western blot assays (Figure 2 A). As shown in Figure 2B, transfection of splenic adherent cells with siRNA directed towards TLR4 but not TLR2 significantly abrogated GSPL-mediated- parasite suppressive effect. The addition of polymyxin B (100 ng/mL) did not alter the GSPL mediated killing of the parasite (Figure 2 B).

Since GSPL did not confer protection in presence of TLR4 Ab or siRNA, we evaluated therapeutic efficacy of GSPL in *LD* infected TLR4 defective C3H/HeJ mice. Sixty days post-infection, *LD* infected C3H/HeJ mice were treated twice at 15 days interval with GSPL (100 µg/dose). GSPL elicited very little protection in the C3H/HeJ mice compared with the vehicle treated mice and the parasite load remained high in the treated mice (Figure 2 C,D). Our findings demonstrate that TLR4 is essential for protective immunity elicited by GSPL.

To ascertain if host responses mediated through TLR4 contribute to parasite clearance in an iNKT independent manner, we used LPS to examine the effect of TLR4 activation on intracellular parasite replication. Sixty days post-infection, *LD* infected BALB/c mice were treated with LPS thrice (5 µg/dose or 10 µg/dose; intraperitoneal injection) on alternate days and animals were sacrificed on days 1, 3 and 12 after the last treatment. LPS treatment did not lead to reduced parasite burden (Figure 2 E,F).

TLR/MyD88-dependent signaling has been implicated as essential for the immune responses against *Leishmania* parasites [17]. To determine the effect of MyD88 on TLR4 mediated protection, BMDCs were either mock transfected or transfected with MyD88 siRNA, infected with *LD* parasites followed by GSPL treatment. GSPL-induced inhibition of amastigote multiplication was also found to be markedly attenuated by MyD88 gene silencing (Figure 2 B). MyD88 silencing was confirmed by western blot assays (Figure 2 A).

### GSPL induces TLR4 expression on splenic adherent cells

**Figure 3:**
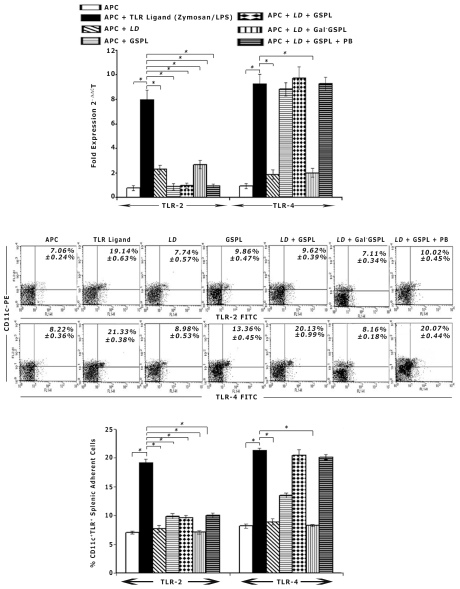
GSPL upregulates TLR4 expression in splenic adherent cells. Splenic adherent cells were infected with *LD* promastigotes (24 h) at 20∶1 parasite-to-splenic cell ratio followed by treatment with 100 µg/mL of GSPL, β-galactosidase treated GSPL (Gal^−^GSPL) or GSPL along with 100 ng/mL Polymixin B (GSPL+PB) for 9 h. TLR expressions were also evaluated in unstimulated splenic adherent cells and adherent cells stimulated with GSPL, Zymosan (TLR2) or LPS (TLR4) for 9 h. TLR expression was evaluated by real-time PCR (A) and flow cytometry (B) for individual cover slips. Bar graphs in B show the percent of CD11c+ cells expressing TLR2 or TLR4 in splenic adherent cells. The results are representative of three independent experiments. Error bars represent mean ± SD, n=3. * p<0.0001; paired two-tailed Student's t-test.

To determine if GSPL could modulate TLR expression, we analysed the expression of TLR2, and 4 in *LD* infected GSPL treated APCs by real-time-PCR. Stimulation with the TLR ligands Zymosan (TLR2) and LPS (TLR4) induced increased expression of the corresponding TLRs. GSPL treatment significantly increased TLR4 expression but not TLR2 expression, in both *LD* infected and uninfected APCs (Figure 3 A).

We next assessed the expression of TLR proteins by flow-cytometry. Significant numbers of CD11c^+^ spleen cells expressed TLR2 and 4. Compared to *LD* infected cells, there was a 2.45-fold increase in the number of CD11c^+^TLR4^+^ cells on GSPL treatment in *LD* infected APCs. There was also a 1.63-fold increase in the number of CD11c^+^TLR4+ cells on GSPL treatment in the uninfected APCs, where as there was no significant change in the expression of CD11c^+^TLR2^+^ cells (Figure 3 B,C).

### Induction of IL-12 on GSPL treatment

**Figure 4:**
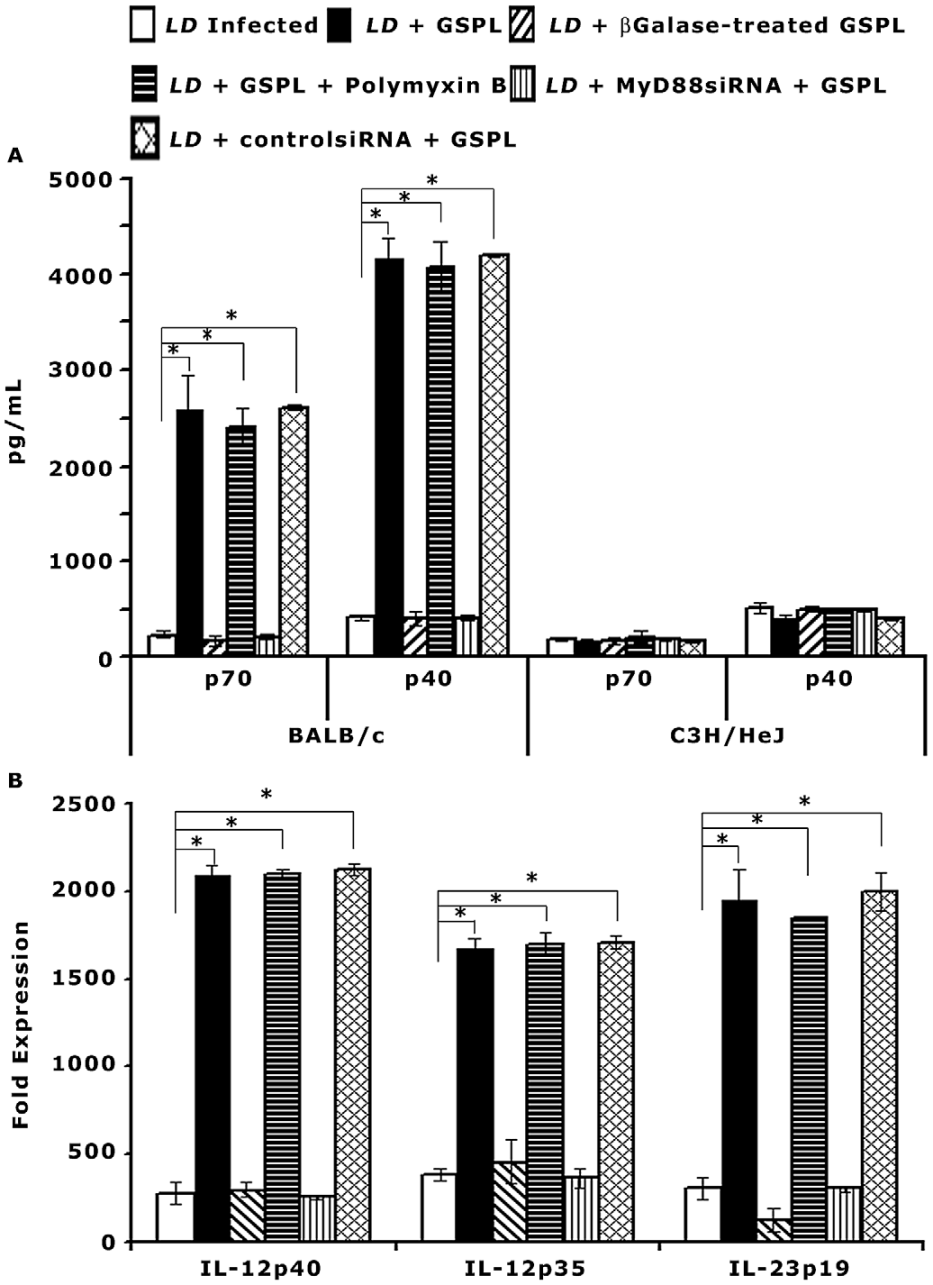
Induction of iNKT cell mediated IL-12 production by splenic adherent cells. Sixty days *LD* infected animals were treated with GSPL as described in the legend of Figure 1. (A) Animals were sacrificed 15 days after the last treatment and iNKT cells isolated from individual experimental animals (WT BALB/c and C3H/HeJ mice) were mixed with 1∶10 of autologous splenic adherent cells (1×10^6^ adherent cells) and were cultured in 24-well culture plates containing 100 µg/mL GSPL for 24 h. Culture supernatants were assayed for the concentration of IL-12p70 and IL-12p40 in ELISA. The results are representative of three independent experiments and data shown are means ± SD; *p<0.0001 versus corresponding infected control; Student's t-test. (B) mRNA expression of IL-12p40, IL-12p35, and IL-23p19 in the spleen of each GSPL treated *LD* infected animals was evaluated individually by real-time PCR. The fold up-regulation of mRNA post-GSPL treatment was calculated by normalizing the amount of cytokine mRNA with the housekeeping gene GAPDH, and comparing results from treated to infected; *p<0.0001; paired two-tailed Student's t-test. (means ± SD (n=3) of one from three independent experiments are shown).

Stimulation of TLR4 induces DC maturation and strong Th1-type responses through release of IL-12 [18]. iNKT cells isolated from experimental animals were adjusted to 1×10^5^ cells/mL and mixed with 1∶10 of autologous splenic adherent cells (1×10^6^ adherent cells). To determine the pattern of adherent cell composition, we performed flow cytometric analysis (Figure S4). Adherent cells were composed of 72% Cd11b^+^ cells, 7% Cd11c^+^ cells and 18% Cd11b^+^Cd11c^+^ cell populations. The purity of the sorted NKT cells averaged greater than 99 percent (Figure S5 Aiii). To determine whether IL-12 secretion following iNKT cell activation correlates to *in vivo* induction of Th1 and Th2 cells, APC were cultured with purified iNKT cells as described by Maruo *et. al.* [19]. Culture supernatants were collected after 24 h and IL-12p70 and IL-12p40 was measured by ELISA. There was ∼10.9 and 10 fold increase in the production of IL-12p70 and IL-12p40 respectively in the GSPL treated *LD* infected BALB/c mice compared to the infected control animals (Figure 4 A). IL-12p70 and IL-12p40 secretion in the GSPL treated C3H/HeJ mice were appreciably lower compared to the cytokine levels in wild type (WT) BALB/c mice. There was no appreciable change in IL-12p70 or IL-12p40 expression in presence of polymyxin B (100 ng/mL). This result rules out the possibility that there was any LPS contamination in GSPL.

Release of IL-12 by dendritic cells (DC) activated by TLR ligation is dependent on MyD88 signaling [20]. GSPL-induced IL-12 production was found to be markedly attenuated by MyD88 gene silencing (Figure 4 A). There was 11 and 10 fold increase in the production of IL-12p70 and IL-12p40 respectively by APCs that were transfected with non-silencing siRNA control (Figure 4 A).

Since there was an increase in IL-12p40, we assessed the expression of the IL-12 and IL-23 specific chains IL-12p35 and IL-23p19 by real-time PCR. There was significant increase in the expression of both IL-12p35 and IL-23p19 in GSPL treated BALB/c mice (Figure 4 B).

### Induction of host protective Th1 immune response on GSPL treatment

**Figure 5:**
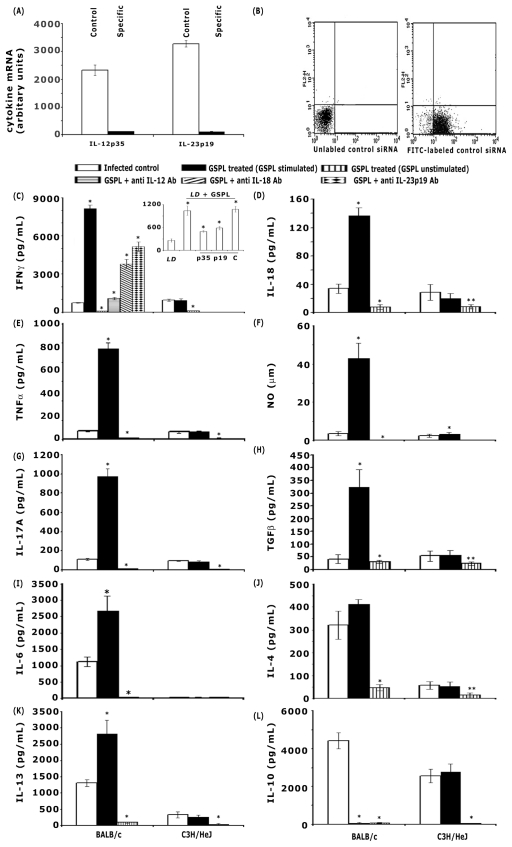
GSPL treatment induces iNKT cell mediated Th1/Th17 cytokines in BALB/c. iNKT cells isolated from experimental animals were mixed with autologous splenic adherent cells as described for Figure 4. Cells were stimulated with GSPL at 100 µg/mL for the time periods as mentioned in the text. Cytokines and NO in spleen cell culture supernatants of five individual animals from each group were determined by ELISA and Greiss assay method, respectively. (A) Splenic adherent cells were transfected with siRNAs specific to IL-12p35 or IL-23p19 (cytokine). A control group was transfected with control siRNA (c). Twenty-four hours after transfection, cells were recovered and their cytokine levels assessed by real-time PCR. GAPDH in total proteins was used as loading controls. Data is the representative of three separate experiments. (B) Transfection efficacy was quantified using FITC-labeled scrambled siRNA control and flow-cytometry. (C) Requirement of IL-12 and related cytokines in IFN-γ production by collaboration between V_α_14^+^ NKT cells and APCs. Anti IL-12p70, anti IL-18 and anti IL-23p19 Abs or isotype controls (data not shown) were added to parallel cultures and IFN-γ production was assessed in the supernatant by ELISA. Adherent cells were transfected with IL-23p19, IL-12p35 and control siRNA and IFN-γ production was assessed (C, insert). D, IL-18; E, TNF-α; F, NO; G, IL-17A; H, TGF-β; I, IL-6; J, IL-4; K, IL-13 and L, IL-10 expressions. Data represent the mean ± SD for five animals per group. (*p<0.0001 versus corresponding infected control; paired two-tailed Student's t-test). Data are representative of three experiments.

For IFN-γ, IL-18, TNF-α and NO estimation, iNKT cells isolated from experimental BALB/c mice were mixed with autologous splenic adherent cells as described above. Supernatants from GSPL stimulated and un-stimulated cultures were collected after 48 h for IL-18 and 72 h for IFN-γ, TNF-α and nitric oxide (NO), and the levels of cytokines were measured by ELISA and NO by the Griess Reagent. Cells were also analyzed by intracellular cytokine staining and NO staining with 4,5-diaminofluorescein diacetate. No cytokines were detected without antigenic stimulation of spleen cell cultures. Cytokine profile at the protein level was assessed by ELISA. Compared to the *LD* infected animals, there was a 10, 4.5, 10.3 and 11.4 fold up-regulation of IFN-γ, IL-18, TNF-α, and NO respectively in the cured BALB/c mice, where as the cytokine levels were appreciably lower in the GSPL treated C3H/HeJ mice (Figure 5, C–F). Intracellular cytokine levels as measured by flow cytometry revealed a similar increase of IFN-γ, IL-18, and TNF-α in spleen cells of GSPL-treated infected animals (Figure S5). To specifically identify iNKT cells, we gated on cells that doubly stained with fluorescent PE–CD1d–α-GalCer tetramers and PE-Cy5-anti-TCRβ (Figure S5, C, gate R5) and analyzed intracellular FITC-labeled cytokines in the gated populations. Consistent with our ELISA studies, and intracellular cytokine profiles, transcript levels for IFN-γ, IL-18, TNF-α, and iNOS in spleen cells of GSPL-treated infected animals showed similar results as assessed by real-time PCR (Figure S6, A-D). IFN-γ and NO are important mediators of antileishmanial immunity. IL-18 has been demonstrated to act in concert with both IL-12 and IL-23 to enhance IFN-γ production mediated by either NKT or NK cells [21]–[23]. To investigate if IL-12, IL-18, or Il-23 enhanced IFNγ production, we examined the effect of neutralizing anti IL-12, anti IL-18 or anti IL-23 Abs on IFNγ production. Significantly lower levels of IFN-γ were produced when cultures of BALB/c cells were incubated with either anti IL-12p70, anti IL-18 or anti IL-23p19 antibody (10 µg/mL) (Figure 5 C, Figure S5). Treatment with control IgG did not have any effect on IFN-γ secretion (data not shown).

To define the physiologic role of IL-12, IL-18 and IL-23 in GSPL mediated protection, we further examined the effect of neutralizing anti-IL-12, anti IL-18 and anti IL-23 Abs on *LD* infected mice. Administration of the Abs significantly abrogated the protective efficacy of GSPL (Figure S7).

Since, the C17.8 antibody has the potential to recognize the p40 subunit of both IL-12 and IL-23 (composed of p19 and p40) we knocked down IL-12p35, and IL-23p19 in BMDC with IL-12p35 and IL-23p19 specific siRNAs respectively, infected the BMDCs *in vitro* for 48 h before treating with 100 µg/mL GSPL. Cytokine silencing was confirmed by real-time-PCR (Figure 5 A). The data were normalized using the housekeeping gene GAPDH. IFN-γ produced by *LD*-infected GSPL treated control BMDC (un-transfected) was 1039.7±123.10 pg/mL. Transfection of BMDC with IL-12p35 or IL-23p19 specific siRNAs resulted in a significant reduction in IFNγ production (Figure 5 C, insert). Transfection with scrambled siRNA did not have any effect on IFN-γ secretion. Transfection efficacy was determined using FITC-labeled scrambled siRNA control. The transfection efficiency was quantified by flow-cytometry. As shown in Figure 5B, FITC-labeled control siRNA successfully transfected >93% of the cells. Since IFN-γ is needed for optimal induction of NO, a predicted consequence of IFNγ down-regulation was a significant reduction in NO production (data not shown).

### Th2 and Th17 related cytokines

GSPL treatment significantly enhanced the expression of IL-17A, TGF-β and IL-6 in the *LD*-infected BALB/c mice. This was reflected in a 4.2, 4.7 and 2.4 fold increases in IL-17A, TGF-β and IL-6 protein detected by ELISA in culture supernatants (Figure 5 G–I). Though IL-4 expression was comparable in the infected and cured groups (Figure 5 J, IL-13 level was higher in the cured group of BALB/c mice (Figure 5 K). The elevated IL-10 expression in the infected BALB/c mice declined in the cured group of mice (Figure 5 L). There was no appreciable change in cytokine expression in the GSPL treated and untreated *LD*-infected C3H/HeJ mice (Figure 5, G–L). Similar results were obtained when the intracellular cytokine and mRNA transcripts levels of the infected and cured groups was compared (Figure S5, S6).

### IL-17^+^-NKT cells in GSPL mediated cure

**Figure 6:**
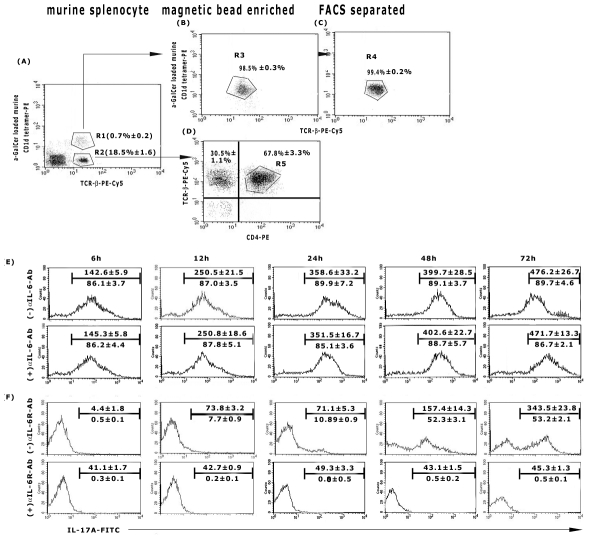
Kinetics of IL-17A production by iNKT cells of cured mice. Sixty days *LD* infected animals were treated with GSPL as described in the legend of Figure 1. Animals were sacrificed 15 days after the last treatment. NKT cells from spleens of individual experimental BALB/c (A) mice were identified as α-GC/CD1d tetramer^+^TCRβ^+^ cells and after enrichment by magnetic cell sorting (B) were further purified by FACS sorting (C). The NKT^−^ populations were identified as TCRβ^+^CD4^+^ cells (D). Isolated (E) iNKT cells or (F) iNKT depleted cell populations (R5, TCR-β^+^CD4^+^) were co-cultured with 1∶10 of autologous splenic adherent cells as described for Figure 4. Cells were stimulated with GSPL at 100 µg/mL, ± IL-6 Ab (E) or ± IL-6R Ab (F) for the time periods indicated and intracellular IL-17A was assessed using FACS analysis. Numbers above the horizontal bar indicates the mean fluorescence intensity and number below the bar indicates the percentage of IL-17 positive cells in the gated populations. Results show mean ± SD of three individual mice per group; paired two-tailed Student's t-test. Results of one from three independent experiments are shown.

It has been suggested that a rapid innate IL-17 production by NKT cells precedes the adaptive IL-17 response [24]. To explore this probability, GSPL stimulated spleen cells from infected and GSPL treated cured animals were fractionated into CD1d-αGalCer tetramer^+^ iNKT cells and iNKT cell depleted populations. iNKT cells were purified using MACS beads followed by FACS sorting. To specifically identify iNKT cells, we gated on cells that doubly stained with fluorescent PE–CD1d–α-GalCer tetramers and PE-Cy5-anti-TCRβ (Figure 6 A–C). We gated further on the CD1d-α-GalCer^−^ PE-Cy5-anti-TCRβ^+^ PE-CD4^+^ (Figure 6 D) and analysed IL-17A production in both the gated populations. Kinetics of IL-17A production in cultured supernatants of iNKT cells or iNKT^−^ CD4^+^ TCRβ^+^ cell populations (1×10^5^ cells/mL) co-cultured with 1∶10 of autologous splenic adherent cells revealed that there was significant IL-17A production by NKT cells from cured mice within 6 h that reached a plateau by 24 h (Figure 6 E), while within iNKT^−^CD4^+^TCRβ^+^ populations in these mice, though there was very little IL-17A production up to 24 h, IL-17 production markedly increased thereafter (Figure 6 F). Though mouse T_H_17 cells have been reported to require IL-6 and transforming growth factor-β (TGF-β) for lineage commitment and IL-23 for maintenance, the early production of IL-17 by NKT cells is independent of IL-6 [24]. Consistent with an expandable role for IL-6 in the expression of IL-17 by NKT cells, IL-6 neutralization (20 ng/200 µL) did not effect the expression of IL-17A in NKT cells (Figure 6E), though IL-17A secretion from iNKT^−^ CD4^+^ cells was almost completely inhibited when the cells were pretreated with an anti-mouse IL-6R Ab (1 µg/mL) (Figure 6 F). Treatment with isotype control Abs did not have any effect on IL-17A production by T cells (data not shown). A similar IL-17A expression profile of iNKT and iNKT^−^CD4^+^ TCRβ^+^ cells was also reflected in terms of protein secretion as determined by ELISA (Figure S 8).

### TLR4/IL-23p19 mediated IL-17 induction

Based on the knowledge regarding the role of TLR4 in IL-23p19 mediated IL-17 expression by iNKT cells [25], we wanted to assess whether TLR4 signaling was critical for IL-23p19 mediated IL-17A production in GSPL treated *LD*-infected BALB/c mice. iNKT cells (1×10^5^ cells/ml, isolated from experimental animals) co-cultured with 1∶10 of autologous splenic adherent cells were stimulated with GSPL (100 µg/mL). There was an up-regulation of IL-17A protein expression from 603.67±22.94 pg/mL in infected group of animals to 5209.33±316.09 pg/mL in the cured group. Ab neutralizing anti-IL-23p19 (10 µg/mL) abrogated IL-17A expression in GSPL-pulsed iNKT cell-autologous adherent cell co-culture from cured group of animals (Figure S9). Silencing of IL-23p19 expression in adherent cells by IL-23p19 specific siRNA transfection before setting up adherent cell-autologous non-adherent cell (iNKT cells) co-culture also abated IL-17A secretion (Figure S9). Isotype control or scrambled siRNA transfection did not have any effect on IL-17A expression.

**Figure 7:**
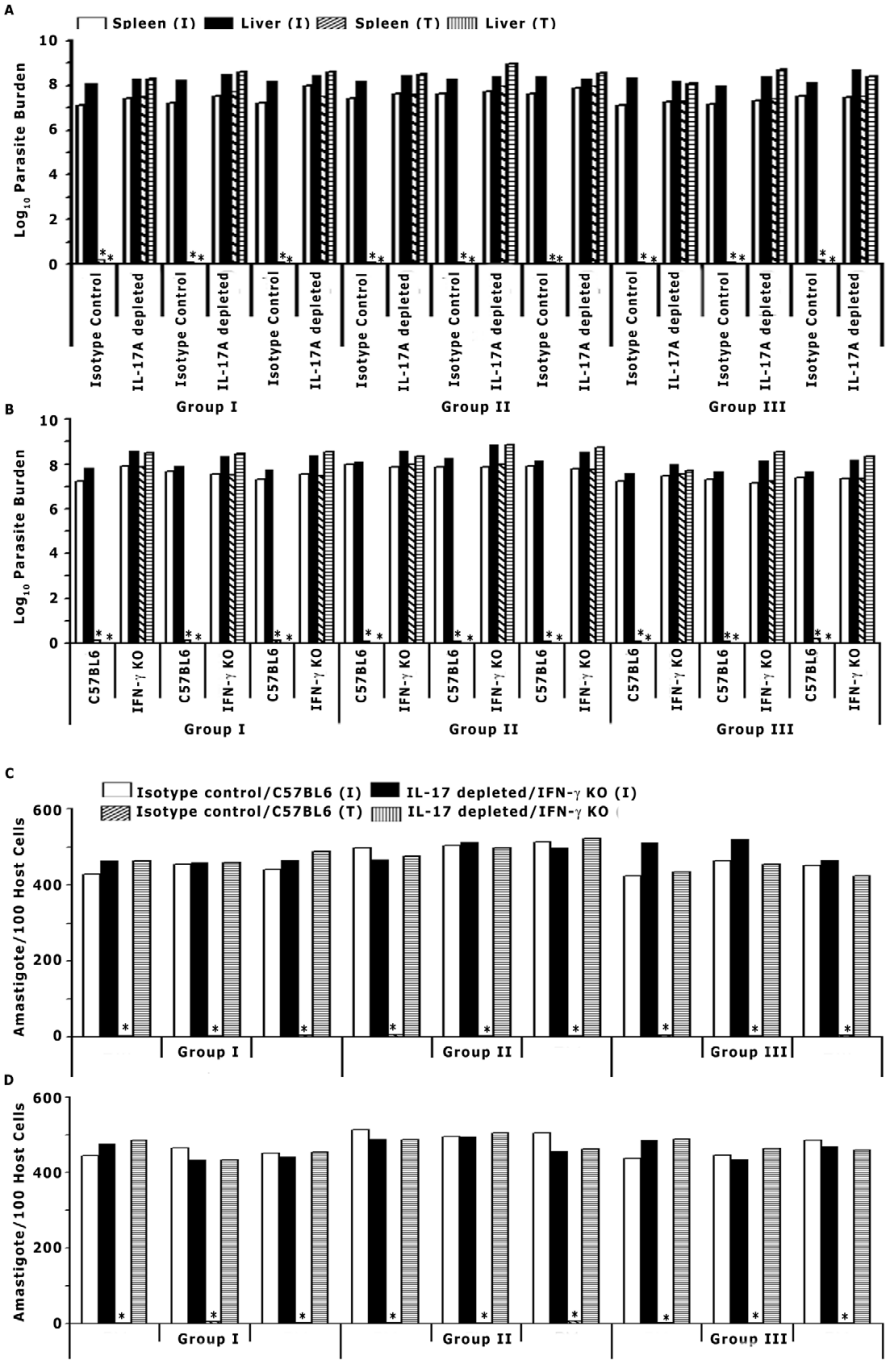
GSPL mediated cure requires the dual activation of the IFN-γ and IL-17 signaling pathways. (A) Sixty days *LD*-infected BALB/c mice were treated with anti IL-17A Ab or isotype control Ab on days −1, 0, and +1 of GSPL treatment. The parasite burdens in (A) liver, spleen, and (C) bone marrow was then determined at 15 days after the last treatment. (B) Sixty days *LD*-infected WT C57BL/6 (WT) and IFN-γ KO mice were treated with GSPL and the parasite burdens in liver, spleen, and (D) bone marrow was then determined at 15 days after the last treatment. Experiments were repeated three times with three mice per group. The data are presented from individual mice of all three groups. *p<0.0001 versus corresponding infected control; paired two-tailed Student's t-test. T, GSPL treated; I, *LD* infected.

These results suggested that IL-17A may play a role in GSPL therapy against experimental VL. We therefore examined the effects of IL-17A depletion on the development of GSPL mediated cure in mice given an experimental *LD* infection. *LD* infected animals were treated with neutralizing antibodies against IL-17A. IL-17A depleted animals had significantly higher organ parasite burden compared to mice treated with the isotype control antibody (*p*<0.0001) at day 15 post-treatment (Figure 7 A,C). These results suggest that IL-17 may be important for optimal protective immune responsiveness during GSPL therapy.

### Role of IFN-γ during GSPL mediated cure

From the results it appeared that GSPL conditioned spleen cells drive concurrent IFN-γ and IL-17A. To assess the direct role of IFN-γ in GSPL mediated protection, *LD* infected BALB/c splenic macrophages adhered on cover-slips (4578±115.53 parasites/1000 adherent cells) were treated with GSPL in presence and absence of anti IFN-γ Ab. While single exposure to 100 µg/mL GSPL for 24 h post-infection, reduced the intracellular parasite load to virtually zero (4.52±1.0 parasites/1000 adherent cells), 1 h pre-treatment with 20 ng anti IFN-γ significantly abrogated the GSPL mediated protection (4342.6±173.44 parasites/1000 adherent cells). Further evidence for the critical role for IFN-γ in the control of *LD* infection comes from the demonstration that GSPL treatment failed to cure infection in IFN-γ knockout (KO) mice (Figure 7 B,D). Treatment of *LD* infected C57BL/6 WT mice with GSPL (100 µg/dose) resulted in complete absence of promastigotes in the serially diluted spleen cell cultures till 21 days of observation (Figure 7 B) in 4 out of 5 animals, while there was no parasite detected in the liver of the animals treated with GSPL. Thus, IFN-γ deficiency completely abrogated the GSPL mediated protection.

### Role of CD40-CD40L interaction in GSPL immunotherapy

**Figure 8:**
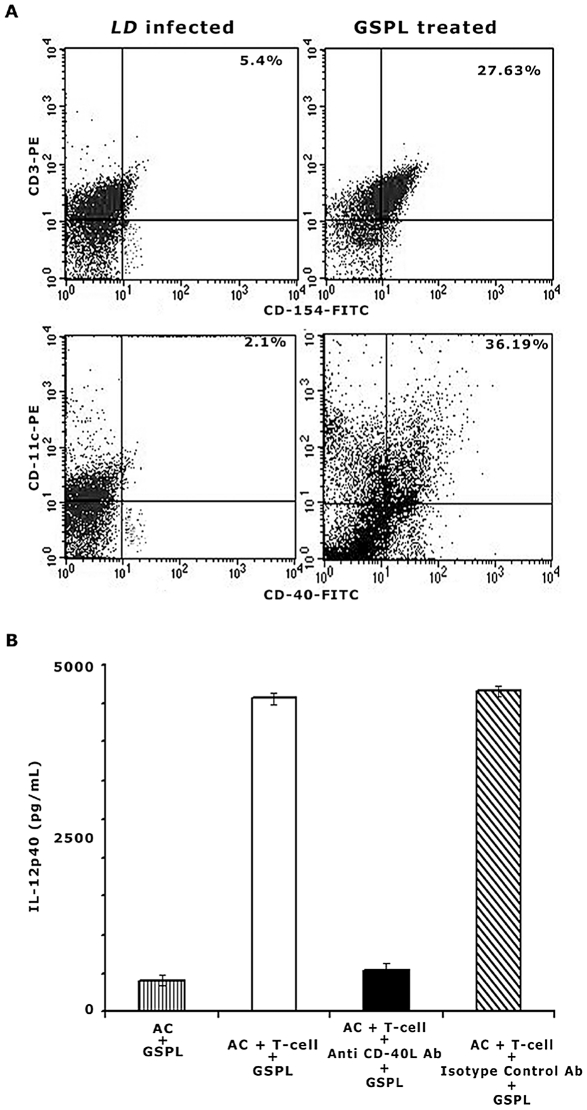
CD40-CD40L ligation augments IL-12 production in GSPL immunotherapy. (A) Comparison of CD40 and CD40L expression in CD11c+ APC and CD3+ T cells respectively between infected and cured animals. Sixty days *LD* infected animals were treated with GSPL as described in the legend of Figure 1. Animals were sacrificed 15 days after the last treatment and expression of CD40 and CD40L was studied in CD11c+ APC and CD3+ T cells respectively (B) Splenocytes from infected and cured animals were stimulated with GSPL. Anti CD40L or isotype controls were added to parallel cultures and IL-12p40 was assessed in the culture supernatants by ELISA. IL-12p40 was also measured in the culture supernatants of splenic adherent cells (AC) in absence of T-cells. The results are representative of three individual mice per group and data shown are means ± SD; *p<0.0001 versus corresponding infected control; paired two-tailed Student's t-test. Results of one from three independent experiments are shown.

IL-12 production as a result of DC-iNKT cell interaction requires ligation of CD40 by CD40L. To determine the involvement of CD40-CD40L (CD154) ligation, we analyzed the expression of CD40 and CD40L on DC and T cell surface respectively from GSPL treated and untreated *LD* infected BALB/c mice. On GSPL treatment, CD40 and CD154 expression increased on the surface of DC (36.19%) and T (27.63%) cells respectively, as compared to the *LD* infected animals (2.1% and 5.4% respectively) (Figure 8A). As shown earlier, GSPL treatment resulted in an increase in IL-12 production. Blocking CD40-CD40L interactions with anti CD40L mAb inhibited IL-12 production by splenocytes from cured animals (Figure 8 B). Isotype matched control antibody showed no effect on IL-12 production. Adherent cells lacking T cells isolated from the cured animals did not produce IL-12p40 (<45 pg/ml) on *in vitro* stimulation with 100 µg/mL GSPL. These results indicated that the CD40-CD40L dependent IL-12 production in splenocytes occurred as a result of a direct cognate interaction between T cells and adherent cells.

## Discussion

Glycolipid activated iNKT cells are capable of producing both Th1 and Th2 cytokine responses. The stimulatory Th1/Th2 balance is dictated by the presence of other maturation stimuli simultaneously acting on DCs [26]. Although, interaction of the same iNKT cells with the DCs in the presence of simultaneous TLR4 stimulation significantly enhances proinflammatory DC maturation and IL-12 secretion [26], the therapeutic implication of this phenomenon has not been exploited.

**Figure 9:**
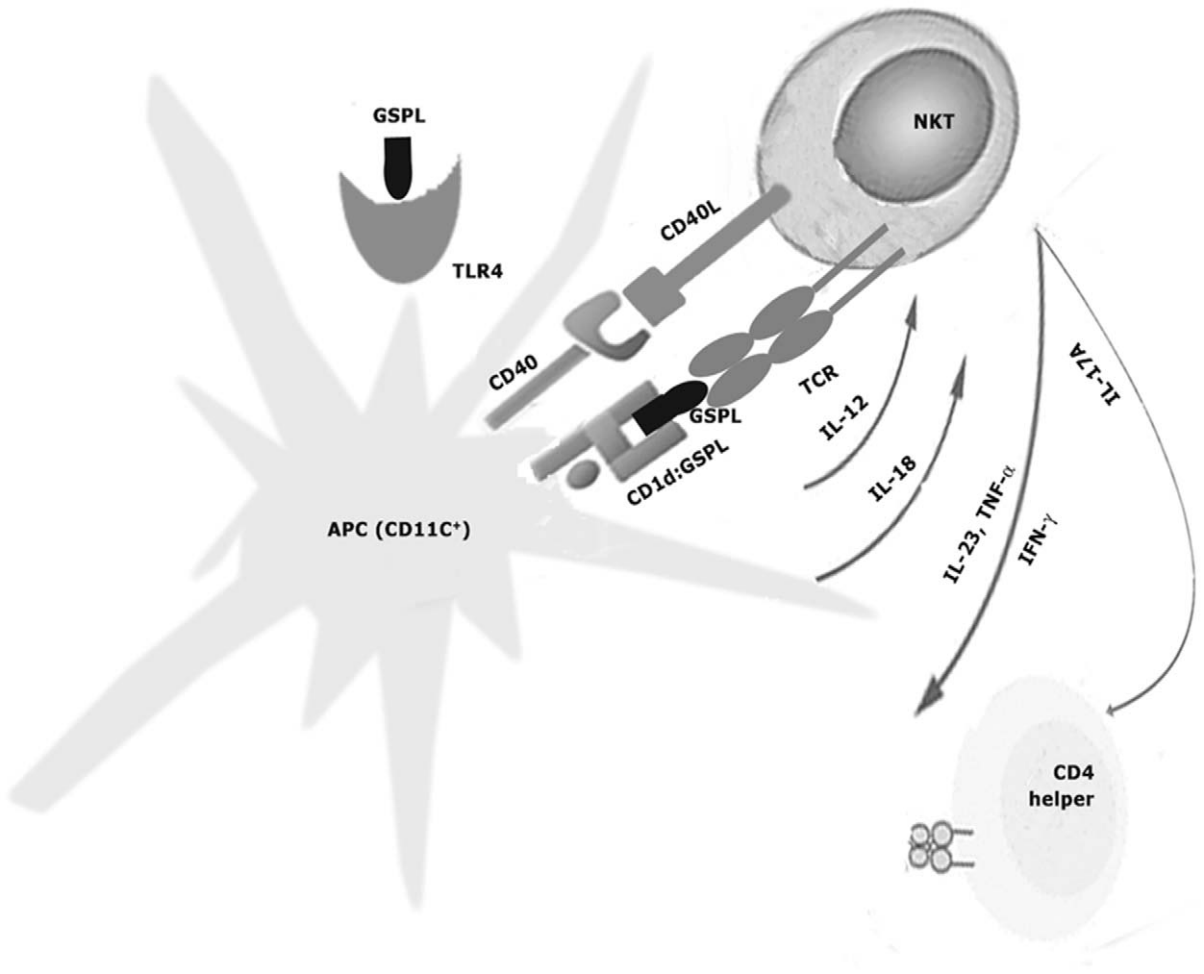
Role of NKT-cells in GSPL mediated therapy. Recognition of GSPL via TLR4 results in IL-12 release by CD11c^+^APCs. GSPL recognized in the context of CD1d molecules stimulates iNKT-cells through their invariant T cell receptor (TCR). Upon activation, NKT cells rapidly secrete cytokines such as IFN-γ and IL-17A, and together with CD40-CD40L interaction, induce activation of CD11c^+^APCs. Thus, GSPL may activate NKT-cells via CD1d-dependent and TLR-4-dependent mechanisms and these mechanisms can co-operate to enhance the efficacy of GSPL mediated immunotherapy by augmenting the NKT driven immune responses.

Previous studies have indicated a rather controversial role of the CD1d-restricted NKT cells in VL. Studies in CD1d-deficient BALB/c mice suggested that NKT cells were required for efficient control of hepatic *LD* infection [27], while, CD1d-restricted NKT cells have been reported to play only a minor physiological role in experimental VL in C57BL/6 mice [28]. Previous work from our group has shown that stimulation of NKT cells by GSPL requires the presence of CD1d [11]. Here we show that stimulation of NKT cells and TLR4 by *Leishmania* glycolipid Ag GSPL leads to the production of IFN-γ and IL-17A and the subsequent clearance of organ parasite burdens in a mouse model of experimental VL (Figure 9). In the BALB/c mouse model infected i.c. with *L. donovani* it has been reported that the liver parasite load reaches a maximum around 2-month post-infection period following which it starts declining, while parasite load in the spleen increases up to 4 months and thereafter the animals maintain chronic infection with decreased parasite load for many months, if not for life. [29]. On the other hand, VL induced by intravenous inoculation of parasites results in a faster clearance of liver parasite burden that peaks at 4 wks p.i. [29]. In the early stage of infection, in absence of activated T cells, number of parasites in the liver reaches a peak. With the acquisition of a granulomatous response at the later stage of infection, the liver parasite burden decreases [30]. In contrast to the liver, 50% of the ingested parasite inoculum is killed by the marginal zone macrophages within the first 24 h. Failure to activate intrinsic leishminicidal mechanism together with the inability to develop granulomatous immune effector responses contribute to the failure of the spleen to resolve VL [30]. The sixty days infected murine model of leishmaniasis was used since we reasoned that an efficient therapeutic agent should be able to control the splenic parasite burden.

The importance of the membrane-associated *LD* glycolipid Ag GSPL comes from previous results, demonstrating that GSPL 1) can induce Th1 cytokines and NO generation in Mφ [12], and 2) can stimulate robust IL-2 production in Vα14Jα18 NKT hybridoma cells [11]. Since lack of anti-*Leishmania* CMI has been considered a hallmark of VL [31], parasite antigens that can control Th2 expansion and promote the predominance of a Th1 type of response should be potential candidates for specific immunotherapy. Two subcutaneous (s.c.) inoculations of GSPL (100 µg each) 15 days apart completely cleared intracellular *LD* parasites in BALB/c mice. Successful therapy should not only kill the intracellular amastigotes, but should also prevent post-treatment relapse. GSPL treated animals remained parasite-free up-to 12 months post-treatment.

The ability to switch between type1 (IFN-γ) and type 2 (IL-4) cytokines emphasizes the immunological regulatory role that the NKT cells play. The mechanism by which NKT cells select the cytokines they secret are not well characterized. Cytokine response induced by stimulated DCs is influenced by pattern recognition [32]. Pathogen-associated molecular patterns (PAMP) that directly stimulate DCs via TLRs act together with signals from activated iNKT cells to influence the quality of immune responses induced [33]. Terminal β-(1–4)-galactose residues in glycans have been identified as the ligand that can induce IFN-γ via TLR signaling [8], [34]. Presence of terminal β1,4 linked galactosyl residues in GSPL has been previously reported [9]. We have reported previously that GSPL stimulates iNKT cells [11]. Conclusive evidence that terminal β-(1–4)-galactose residues are involved in GSPL mediated protection was provided by the observation that enzymatic removal of the terminal galactose completely abrogated protective efficacy of GSPL.

TLR4 expression is low in the macrophages and DCs and has been shown to be regulated by inflammatory cytokines [35]. As TLRs are instrumental in both launching innate immune responses and influencing adaptive immunity [36], regulation of TLR expression may be important in the pathophysiology of VL. Gene knockout studies in mice have suggested that TLR signalling is essential for the immune responses against *Leishmania* parasites [17]. A number of *in vitro* and *in vivo* studies have already documented the importance of various TLRs in host defence against different forms of leishmaniasis [37]–[43]. Further, TLRs have the potential to act as therapeutic targets. In the recent years, TLR agonists are being developed for the treatment of cancer, allergies and viral infections and as adjuvants to enhance immune responses against tumors and infectious diseases [44]. GSPL induced the expression of TLR4 but not 2 in infected BMDCs and silencing of TLR4 markedly attenuated the leishmanicidal activity of GSPL, thereby suggesting the importance of downstream TLR4-dependent signaling in anti-leishmanial effector response. To further substantiate the role of TLR4 in protection against VL, we compared the protective efficacy of GSPL in *LD* infected TLR4 defective C3H/HeJ and WT BALB/c mice. GSPL mediated protection was completely abrogated in C3H/HeJ TLR4 mutant mice. To address the question whether iNKT independent TLR4 dependent immune response is essential for parasite clearance, *LD* infected BALB/c mice were treated with LPS. However, since LPS could not substitute for GSPL, it appears that GSPL mediated activation of TLR4 is required to bring about the protection seen.

Resistance against *Leishmania* infection remains largely associated with an IL-12 induced type-1 response [45]. TLR signals APCs to produce high levels of IL-12 [46], [47]. In our study IL-12 was completely down-regulated in the AG83- infected mice, whereas high levels of IL-12p40 and IL-12p35 mRNA transcripts and IL-12p70 and IL-12p40 proteins were found in GSPL treated cured BALB/c mice. Very little IL-12 could be detected in the C3H/HeJ mice. Release of IL-12 by DCs activated by TLR ligation is dependent on MyD88 signaling [20]. Inhibition of MyD88, strongly inhibited GSPL mediated parasite suppressive effect in infected BMDCS.

Interaction of PAMPs with Mφs and DCs via TLRs results in a type 1 like response [30], [48], [49]. The cooperative stimulation of TLR and iNKT cells resulted in Th1 skewing on GSPL immunotherapy. There was an up-regulation of the type 1 cytokine IFN-γ, IL-12, IL-18 and IL-23 with a concomitant decrease in the disease promoting IL-10. Besides IL-12, cure is associated with strong IFNγ responses in the absence of IL-10 [50]. Evidence for the critical role for IFN-γ in the GSPL mediated control of *LD* infection came from the demonstration that IFN-γ knockout (KO) mice failed to cure infection. IFN-γ production by NKT cells is a consequence of the synergistic action of IL-18 with IL-12, or IL-23 produced by PAMP stimulated APC [23]. Our results indicated that APCs after binding of PAMPs became primed to subsequently produce large amounts of IL-12, IL-18, and IL-23 and thus amplified IFN-γ production. This conclusion is supported by the finding that IFN-γ production decreased in presence of anti IL-12, anti IL-18 and anti IL-23 Ab. IFN-γ derived from *i*NKT cells inhibits the growth of intracellular microbes by stimulating infected APCs to synthesize NO [51]. IL-12 and IL-18 augment this response. There was 11.4 and 13.5 fold increase in the expression of NO and iNOS transcript respectively in the cured BALB/c mice in comparison to the infected animals.

Although IL-4 and IL-13 are associated with development of type 2 immune responses in models of cutaneous leishmaniasis [52], [53], there are many conflicting reports of both IL-4 and IL-13 having opposite roles to play in VL [54]–[56]. Though IL-4 production was comparable in the infected and cured mice, there was a 2 fold increase in IL-13 production in the GSPL treated BALB/c mice.

In addition to classical Th17 cells, NKT cells also produce IL-17 [24], [57]. Early production of IL-17A by CD1d-αGalCer-tetramer^+^TCR-β^+^ iNKT cells from cured animals validated the earlier findings that innate IL-17 production by NKT cells is rapid and precedes the adaptive IL-17 response [24]. In addition, *in vivo* GSPL treatment also produced TCR-β^+^CD4^+^ T cells capable of producing IL-17A. Early production of IL-17A by iNKT cells was independent of IL-6, while CD4+ T cells produced IL-6 dependent IL-17A. This was in agreement with the previous finding that alpha-galactosylceramide stimulated naive IL-6(−/−) splenocytes produces normal amounts of IL-17 during the first 24 h of culture [24]. IL-23 is known to promote the production of IL-17 by NKT cells mainly in a TLR2/4-dependent manner [25]. IL-17A production by iNKT cells from cured animals decreased in presence of anti IL-23p19 Ab or IL-23p19 specific siRNA.

Interaction of iNKT cells with DCs, in the presence of simultaneous TLR4 stimulation, enhances IL-12 secretion through CD40–CD40L interaction [26]. In the present study we observed that IL-12 production *in vivo* is dependent on CD40∶CD40L ligation in GSPL-treated mice.

Together, these results indicated that TLR4-NKT cell synergism mediated GSPL induced host-protective immunological response in experimental VL. The innate immune component of GSPL immunotherapy required dual activation of the IL-12/IFN-γ and IL-23/IL-17 signaling pathways. IL-23 driven NKT cells induced IL-17A, while GSPL induced IFN-γ production by NKT cells required the simultaneous TLR receptor signaling through MyD88/CD40-CD40L, and secretion of IL-12.

## Materials and Methods

### Ethics statement

Use of both mice and hamsters was approved by the Institutional Animal Ethics Committee of Indian Institute of Chemical Biology, India (Accreditation Number 147/1999/CPCSEA). All animal experimentations were performed according to the National Regulatory Guidelines issued by CPSEA (Committee for the Purpose of Supervision of Experiments on Animals), Ministry of Environment and Forest, Govt. of India.

### Animals, parasites and animal infection

Four to 6 wk old BALB/c mice or C57Bl/6 mice (irrespective of sex, originally bought from Jackson Laboratory, Bar Harbour, Maine), reared in the Indian Institute of Chemical Biology facility were used, with prior approval of the animal ethics committee of the Institute. C57Bl/6-background IFN-γ KO mice and TLR4 defective C3H/HeJ mice were a kind gift of Prof. A. Surolia (National Institute of Immunology, New Delhi). The cells of IFN-γ KO mice reproducibly did not produce detectable IFN-γ under optimal stimulatory conditions (not shown). Pentavalent antimony-responsive AG83 (MHOM/IN/83/AG83) was used for experimental infection [11]. Parasites were maintained in golden hamsters as previously described [11]. Promastigotes obtained after transforming amastigotes from infected spleen, were maintained in M199 [11]. Animals were infected via the intracardiac (i.c.) inoculation of *LD* promastigotes [16]. Splenic and hepatic parasite burden in infected animals were determined as described [16], and results were expressed as mean parasite number ± standard deviation. For bone marrow, the parasite burden was determined microscopically, as the number of parasites per 1,000 host nuclei in smears.

### Purification of GSPL

GSPL was purified from AG83 promastigote membranes as described [11]. In short, late log phase promastigote membrane (1 g wet weight) was extracted with 19 vol of chloroform∶methanol∶ethyl acetate∶pyridine∶4.5N ammonia∶water (15∶15∶5∶0.5∶0.5∶0.5, v/v; Solvent A). Anionic glycolipids were eluted from a DEAE-Sephadex A-25 column with a gradient of KCl in 0.01 M phosphate buffer, pH 6.4, containing 0.05 N ammonium hydroxide and 0.1% sodium salt of taurodeoxycholic acid. The anionic glycolipids were loaded onto a silicic acid column and the glycophosphosphingolipid (GSPL) was eluted with C∶M (4∶6, v/v) and further purified on a RCA-1-Sepharose 4B affinity column. GSPL was eluted with 0.1 M Galactose in Solvent A. Purity of GSPL was checked by HPTLC developed in three different solvent systems, Solvent B, C and D. (Solvent B, chloroform ∶ methanol∶ 0.25N ammonia in 0.25% KCL(65∶45∶9); Solvent C, pyridine∶ethyl acetate∶acetic acid∶0.25% KCl (36∶36∶7∶21, v/v), Solvent D, 1-butanol∶pyridine∶0.25% KCl (3∶2∶1, v/v). Plates were sprayed with either the diphenyl amine reagent for glycolipids [58] or Dittmer and Lester reagent for phospholipids [59].

### Endotoxin detection in the GSPL preparations

The endotoxin level of 100 mg/L GSPL preparation was less than 0.1 endotoxin units (EU)/mL as measured by chromogenic Limulus amoebocyte lysate (‘LAL’) endpoint assay (QCL-1000; BioWhittaker, MD, USA) following the manufacturer's manual.

### BMDC infection

BMDC was generated from bone marrow progenitors in the presence of rmGM-CSF and rmIL-4 [11]. A total of 10^6^ non-adherent bone marrow cells/ml, collected after passage of marrow from the tibias and femurs of BALB/c mice, were seeded in a 24-well plate in the presence of rmGM-CSF (150 U/ml) and rmIL-4 (75 U/ml) and then cultured for 3 days in a 37°C incubator with a 5% CO_2_ supply. On day 3, nonadherent cells (2.5×10^6^/2 ml/well) were again transferred and supplemented with complete medium and cytokines, and subsequently cultures were fed with rmGM-CSF and rmIL-4 on days 5 and 7. After 10 days, the nonadherent cells expressing CD11c assessed by flow cytometry (data not shown) were collected. During the last 24 h of BMDC culture, the cells were grown in the presence of rmTNF-α (20 ng/ml), providing DC maturation stimulus.

For *in vitro* infection of BMDCs, cells were seeded on glass coverslips inside 6-well culture plates to a final number of 2×10^5^ cells per well. Cells were infected with stationary phase 2^nd^ passage *LD* promastigotes at a parasite/APC ratio of 20∶1. The cells were incubated at 37°C with 5% CO_2_. Following 12 h incubation, non-internalized promastigotes were removed by washing with PBS and cells were incubated for another 36 hours using the same culture media. Forty eight hours parasitized APCs are used throughout the study. Cell viability was assessed using an MTT (3-(4,5-Dimethylthiazol-2-yl)-2,5-diphenyltetrazolium bromide)-based colorimetric assay kit (Roche Applied Science, Indianapolis, IN, USA) according to the manufacturer's instructions. GSPL was dried from solution in organic solvent by rotary-evaporation under reduced pressure and re-dissolved in PBS. After brief sonication, the sample micellar solution was added to the culture medium at the selected concentrations for *in vitro* work. Infected BMDCs were treated with the indicated concentration of GSPL for 24 h, the coverslips with the attached infected cells were removed, washed with saline solution, fixed and stained with Giemsa. The average number of intracellular parasites per 1000 BMDC was calculated by counting the cells per coverslip.

### Phagocytic activity assay

The macrophages were tested for their ability to ingest fluorescein isothiocyanate-labelled Latex beads using the Phagocytosis Assay Kit (Cayman, Ann Arbor, Michigan, USA) according to the manufacturer's instructions. The fluorescence was determined using a fluorescence microscope equipped with filters for detecting excitation and emission at 483 nm and 535 nm, respectively. We ensured that the fluorescence measured accounted exclusively for ingested particles, as the signal potentially generated from any non-internalized bioparticles was quenched by the addition of trypan blue, as supplied by the manufacturer.

### Treatment of BMDC with anti TLR antibody

Infected BMDCs were pretreated with anti-TLR4 monoclonal antibody (with a final concentration of 10 µg/mL; clone MTS510, eBioscience), or an isotype matched control (rat IgG2aκ, eBioscience), for 1 h, then washed, three times, with PBS solution. Subsequent, identical steps were taken with the GSPL treated groups. The anti TLR2 monoclonal antibody group was similarly pretreated with anti TLR2 antibody (10 µg/mL, clone mT2.7, eBioscience) or an isotype matched control (mouse IgG2aκ, eBioscience) for 1 h, prior to treatment with GSPL.

### Therapeutic immunization with GSPL

BALB/c mice were infected with 1×10^7^, 2^nd^ passage *LD* promastigotes in saline through the i.c. route. *LD* infected animals were divided into five groups of 20 animals each. Sixty days p.i., animals were injected twice at 15 days interval with 100 µg GSPL/100 µL PBS through the s.c route. Group I mice received 100 µL of vehicle only. Mice in groups II received β1,4-galactosidase treated GSPL twice at 15 days interval (100 µg/100 µL vehicle, subcutaneous), Group III mice received 100 µL of αgalactosidase treated GSPL twice at 15 days interval (100 µg/100 µL vehicle, s.c.), Group IV received GSPL only while group V received 100 ng/mL polymyxin/dose along with GSPL. Fifteen days after the last injection, animals were sacrificed and hepatic and splenic parasite burden was determined from impression smears following methanol fixation and Giemsa staining. For the spleen and liver, the parasite burden was expressed as Leishman-Donovan units (LDU),of Stauber on Giemsa-stained imprints (LDU=number of amastigotes/1000 cells nuclei x mg organ weight), as well as by the limiting dilution method [11]. A weighed piece of spleen or liver from experimental animal was first homogenized between two sterile frosted glass slides in complete M199 medium and diluted with the same medium to a final concentration of 1 mg/mL. Ten-fold serial dilutions of the homogenized tissue suspensions were then plated in 96-well plates and incubated at 22°C for 2–3 wk. Wells were examined for viable and motile promastigotes at a 3-day interval, and the reciprocal of the highest dilution that was positive for parasites was considered to be the parasite concentration per milligram of tissue. The total organ parasite burden was calculated using the weight of the respective organs.

### LPS treatment

Sixty days *LD* infected mice were treated with 5 µg/dose or 10 µg/dose LPS (intraperitoneal, Escherichia.coli 055:B5, Sigma) on days 0,2 and 4 and animals were sacrificed on days 1, 3 and 12 after the last treatment. Parasite loads of liver, spleen, and bone marrow of individual animals were determined as mentioned above.

### Galactosidase treatment

Galactosidase treatment was performed using 500 µg GSPL. Treatment with α-galactosidase (G8507, Sigma-Aldrich, St.Louis,) was carried out using 90 mU of enzyme and p-nitrophenyl α-D-galactopyranoside as a positive control. Treatment with positionaly specific β1–4 galactosidase (G-0413, Sigma-Aldrich, St.Louis,), was carried out using 9 mU of enzyme and p- nitrophenyl β-D-galactopyranoside as the positive control. For all enzyme treatments both the treated sample and an identical negative control were worked up in tandem using the manufacturer's recommended conditions. The hydrolysates were centrifuged (20000 g, 4°C, 15 min), and the glycosphingolipids (GSL) were precipitated in cold acetone for 24 h at 4°C. The resulting GSL pellets were further purified by preparative TLC and purified compounds were confirmed by TLC. 10 µg of α/β-galactosidase-treated and untreated samples were run on TLC. Plates were resolved using a solvent system of chloroform∶methanol∶0.25N ammonia in 0.25% KCL(65∶45∶9) (v/v). Typically, two chromatograms were developed in parallel on the same HPTLC plate. One was sprayed with the diphenylamine-aniline-phosphoric acid (DPA) spray reagent [60] and the other was transferred to polyvinylidiene difluoride membrane (PVDF) by TLC blotting [61] and overlaid with biotinylated *Erythrina cristagalli* lectin. The plates were then immersed in buffer B (0.01 M Na2HPO4, 0.14 M NaCl, 2% polyvinylpyrrolidone-40 [pH 7.2]) for 1.5 h at room temperature with gentle stirring (20 rpm). Plates were then incubated with biotinylated *E.cristagalli* lectin (10 µg/ml, 24 h, Vector Laboratory), followed by incubation with streptavidin-HRP(2 h, GE Biosciences) and visualized using a luminol-based, light-producing reaction generated with the enhanced chemiluminescent detection reagent for horse-raddish peroxidase (Pierce). A shift in the chromatographic mobility of β-galactosidase-treated GSPL was observed (Figure S1 A, lane3).

### Real-time RT-PCR

Total RNA was isolated from splenic lymphocytes of BALB/c mice according to the RNeasy minikit isolation procedure (QIAGEN), and was individually analysed (5 animals/group) by real-time reverse transcription PCR. Two µg samples of RNA from different experimental groups of mice were first utilized for cDNA synthesis by random hexamers (Invitrogen) using Superscript II (Invitrogen). The synthesized cDNA was subjected to real-time PCR with SYBR Green JumpStart Taq Ready Mix (Sigma) and gene-specific primers in an iCycler PCR detector (Bio-rad) according to the manufacturer's instructions. The primers used for amplification of IL-10, IL-4, IFNγ, TNFα, TGFβ and iNOS were described previously [11]. The primer sequences for p19, p35, IL-6, IL-17, TLR2, TLR4 and GAPDH are given in Table SI. The relative quantization of products was determined by the comparative ΔΔ*C_T_* method. Each gene of interest was normalized to the ß-actin gene and the fold change was compared relative to the normal control.

### Isolation of iNKT cells from mouse spleen

iNKT cells from mouse spleen were isolated according to the method of Benlagha et al. [62]. Mononuclear cells were obtained from spleens of mice by purification over 35% Percoll gradient centrifugation (Sigma-Aldrich). B-cells were depleted prior to iNKT cell enrichment by using CD45R (B220) Microbeads (Miltenyi Biotec). Subsequent isolation of iNKT cell was carried out using a PE-conjugated CD1d tetramer loaded with α-GalCer (Miltenyi Biotec) and anti PE-Microbeads at ice cold temperature following the manufacturer's instructions. The CD1d-tetramer^+^ cells were labelled with PE-αGalCer-Cd1d-tetramer (Proimmune) and PE-Cy5-conjugated anti-mouse TCRβ (clone: H57-597, hamster IgG2a; BD Biosciences) for further αGalCer-Cd1d-tetramer^+^ TCRβ^+^ enrichment by FACS, and are referred to as NKT cells throughout the study. We gated further on the CD1d-α-GalCer^−^ PE-Cy5-anti-TCRβ^+^ PE-CD4^+^ and are referred as the iNKT depleted populations.

### Cytokine analysis by ELISA

In a final volume of 0.2 mL, iNKT cells isolated from experimental animals were adjusted to 1×10^5^ cells/mL and mixed with 1∶10 of autologous splenic adherent cells (1×10^6^ adherent cells) from individual mice (5 animals/group) of different groups of experimental mice and were incubated for 24 h at 37°C with or without 100 µg/mL GSPL. Adherent cells were obtained by incubating 5×l0^7^ spleen cells in 90-mm glass petri dishes in RPMl 1640 supplemented with penicillin/streptomycin, 10% fetal calf serum, and 5×M 2-mercaptoethanol for 3 hr at 37°C. Non-adherent cells were removed, the adherent cells were washed (×2) with warm RPMI-1640 with gentle swirling and adherent cells were gently detached using a rubber policeman. The release of cytokines was measured in the supernatants by commercial ELISA kits (Quantikine M; R&D Systems, Minneapolis, MN, USA; IL-18, e-Bioscience). The detection limit of these assays was <2.5, <4.0, <5.1, <2.0, <2.0, <1.5, <2.5, <4.0, <5.0, <4.6, <1.6, and 10.0 pg/mL for IL-12p70, IL-12p40, TNF-α, IL-4, IL-13, IL-10, IL-17A, TGF-α, IL-6, and IL-18 respectively. Stimulation with PMA (250 ng/mL; Sigma-Aldrich) and anti-mouse CD3 (1 µg/mL; BD Biosciences) or medium only was used as positive and negative controls, respectively. Appropriate isotype controls were also analyzed. The data are represented as the mean ± SD of all the five individual animals per group under consideration.

### Administration of neutralizing antibody

Blockade of IL-18, IL-23, IL-12, and IL-17 was carried with 100 µg of anti-mouse IL-18, IL-23, IL-12, and IL-17 Abs, clone 93-10 (R&D Systems), G23-8 and C17.8 (eBioscience) and TC11-18H10 (Southern Biotech) respectively or an isotype matched control on day −1, 0 and +1 of treatment with GSPL. For CD40L blockade, anti-CD40L mAb MR1 (eBioscience) was added to the cultures. IL-6 was blocked with 20 ng/200 µL anti IL-6 Ab, clone MP5-20F3 (R & D Systems, rat IgG1) and IL-6R was blocked with mouse IL-6R Ab (1 µg/mL, clone D7715A7, eBioscience, rat IgG2b).

### Immunofluorescent staining

For Ag-specific cytokine responses, splenocytes cultured with either GSPL at 100 µg/mL concentration or no antigen (as a negative control) were stained for intra-cytoplasmic cytokine, or surfaced stained for TLR as described [16].

### Transfection of siRNA to splenocytes

For siRNA transfection, cells were transfected with 1 µg of appropriate siRNA or control siRNA according to the manufacturer's instructions (Santa Cruz Biotechnology).

## Supporting Information

Figure S1 **Thin layer chromatogram of GSPL and effect of GSPL on BMDC viability and phagocytic activity.** (A,B) TLC of GSPL and β-glycosidase treated GSPL. Lane 1, GSPL; lanes 2 and 3, α-galactosidase and β-galactosidase treated GSPL respectively. The glycolipids in plate A were visualized with the diphenyl amine reagent for glycolipids. Plate B was transferred to PVDF membrane by TLC blotting and overlaid with biotinylated *Erythrina cristagalli* lectin. A representative chromatogram developed with chloroform∶methanol∶0.25N ammonia in 0.25% KCL(65∶45∶9) is shown. (C) BMDCs were incubated with various concentrations of GSPL (50–400 µg/mL) for 24 h. Cell viability was assessed by the MTT method. (D,E) Phagocytic activity of GSPL treated BMDCs. FITC-coupled latex beads were co-incubated with BMDCs in absence (D) and in presence of GSPL (E). Extracellular beads were removed by extensive washing; the cells were observed under fluorescence microscope. Scale bars, 10 µm.
(TIF)

Figure S2 **Protective effect of GSPL on disease relapse.** *LD*-infected BALB/c mice were treated with GSPL (8 wk p.i.) and were sacrificed every 3 months up-to 12 months and compared to vehicle-treated infected controls. Bone marrow (A), splenic (B) and hepatic (C) parasite burdens were determined as described in *Materials and Methods*. Data represent the means ± SD of 5 animals per group and are representative of three individual experiments. *p*<0.0001 at all time points after infection, compared with respective infected control groups; paired two-tailed Student's t-test
(TIF)

Figure S3 **GSPL mediated protection depends on TLR4.** (A) BMDCs were infected for 48 h with stationary phase 2^nd^ passage *LD* promastigotes at a parasite/APC ratio of 20∶1. Infected BMDCs were treated with 100 µg/mL GSPL for 24 h. Anti TLR2 and anti TLR4 antibodies and isotype controls were added to parallel cultures and intracellular parasite number was determined by Giemsa staining. (B–D) The viability and phagocytic ability of the BMDCs treated with GSPL was assessed as described in legend to Figure S1. (B) Viability of GSPL treated BMDCs in presence of anti TLR2 and anti TLR4 antibodies. The phagocytic activity of BMDCs in absence (C) and presence (D) of GSPL. Experiments were done at least three times each and one set of representative data is shown. Error bars represent mean ± SD, n=3. * p<0.0001; paired two-tailed Student's t-test.
(TIF)

Figure S4 **FACS analysis of splenic adherent cells.** Shown is a representative APC sample. Region R1 defines all Cd11c^+^ cells; region R2 defines all Cd11b^+^ cells and region R3 defines all Cd11b^+^Cd11c^+^ cells. Experiment was done at least three times.
(TIF)

Figure S5 **Intracellular flow cytometric analysis of cytokine profile in GSPL treated LD infected WT BALB/c and C3H/HeJ mice.** Sixty days *LD* infected animals were treated with GSPL as described in the legend of Figure 1. NKT cells from spleens of individual experimental BALB/c (A) and C3H/HeJ (B) mice were identified as α-GC/CD1d tetramer^+^αβTCR^+^ cells (Ai,Bi) and after enrichment by magnetic cell sorting (Aii,Bii;R1,R3) were further purified by FACS sorting (Aiii,Biii;R2,R4). αGalCerCD1d-tetramer^+^TCRβ^+^iNKT cells isolated from experimental animals were mixed with autologous splenic adherent cells as described for Figure 4. Cells were either stimulated with GSPL (+GSPL) at 100 µg/mL or were treated with medium only (−GSPL) for the time periods as mentioned in the text. For the characterization of the cytokine profile, the T cells were then stained using appropriate concentrations of monoclonal antibody directed against the respective cytokines. To specifically identify iNKT cells, we gated on cells that doubly stained with fluorescent PE–CD1d–α-GalCer tetramers and PE-Cy5-anti-TCRβ (C;R5). The gated cells were further analyzed for the expression of intracellular FITC-labeled cytokines. Numbers above horizontal bars represent mean fluorescence intensity and numbers below the bar represents percentage of cytokine positive cells. Anti IL-12p70, anti IL-18 and anti IL-23p19 Abs or isotype controls (data not shown) were added to parallel cultures and intracellular IFN-γ production was determined by FACS (Diii–v)). D,L, IFN-γ; E,M, TNF-α; F,N, IL-17A; G,O, IL-6; H,P, IL-13; I, Q, IL-4; J,R, TGF-β and K, S, IL-10 expressions. Gray lines depict un-stimulated controls and black lines indicate GSPL stimulated cell. Data represent the mean ± SD for five animals per group. Data are representative of three experiments.
(TIF)

Figure S6 **GSPL mediated induction of Th1/Th17 cytokines mRNA.** Gene expression was done by comparative C_T_ method using real-time PCR. Fold change in m-RNA expression profiles of A, IFN-γ; B, IL-18; C, TNF-α; D, iNOS; E, IL-17A; F, TGF-β; G, IL-6; H, IL-4; I, IL-13 and J, IL-10 in splenic lymphocytes of *LD*-infected (infected control) and *LD*-infected-GSPL treated (GSPL treated) mice. Results show mean ± SD of five individual mice per group (*p<0.0001; **p<0.05 versus corresponding infected control; paired two-tailed Student's t-test). Each gene was normalized to the housekeeping gene (β-actin) before fold change was calculated to account for variations between different samples.
(TIF)

Figure S7 **Anti-IL-12p70, anti IL-18, and anti IL-23p19 treatment of LD infected BALB/c mice reduced the anti-leishmanial effect of GSPL.** Sixty days *LD* infected BALB/c mice were injected i.p. on days −1, 0, and +1 of GSPL treatment either with isotype control antibody, anti-IL-12p70, anti IL-18, or IL-23p19. Splenic parasite burden was determined as described in Figure 1. Data represent the mean ± SD of 3 animals per group, and are representative of three individual experiments. *p<0.0001 compared with the *LD* infected control groups; paired two-tailed Student's t-test.
(TIF)

Figure S8 **Kinetics of IL-17A production by iNKT cells of cured mice.** Sixty days *LD* infected animals were treated with GSPL as described in the legend of Figure 1. Animals were sacrificed 15 days after the last treatment. iNKT cells and NKT depleted cell populations from spleens of individual experimental BALB/c mice were isolated as described in legend to Figure 6. Isolated T-cells were co-cultured with 1∶10 of autologous splenic adherent cells as described for Figure 4. Cells were stimulated with GSPL at 100 µg/mL, ± IL-6 Ab or ± IL-6R Ab for the time periods indicated and IL-17A in spleen cell culture supernatants were determined by ELISA. Results show mean ± SD of three individual mice per group; *p<0.0001 versus corresponding Ab treated group; paired two-tailed Student's t-test. Results of one from three independent experiments are shown.
(TIF)

Figure S9 **GSPL induces IL-17, which is dependent on IL-23.** *LD* infected BALB/c mice were treated with GSPL or with vehicle control only. After 15 days, adherent spleen cells co-cultured with autologus iNKT cells were stimulated with GSPL as described in Figure 6, with or without anti IL-23 Ab or isotype controls. IL-17 was estimated in the supernatants by ELISA. IL-23 siRNA transfected adherent splenic cells were similarly cultured in parallel. Data represent the mean ± SD of 3 animals per group, and are representative of three individual experiments. *p<0.0001; paired two-tailed Student's t-test.
(TIF)

Table S1 **Real-time RT-PCR primer sequences.**
(DOC)
