# Supplementary figures and images for: TLR4 and NKT Cell Synergy in Immunotherapy against Visceral Leishmaniasis
Source: PLoS Pathog. 2012 Apr 12;8(4):e1002646. doi: 10.1371/journal.ppat.1002646 (PMC3325212; doi:10.1371/journal.ppat.1002646)

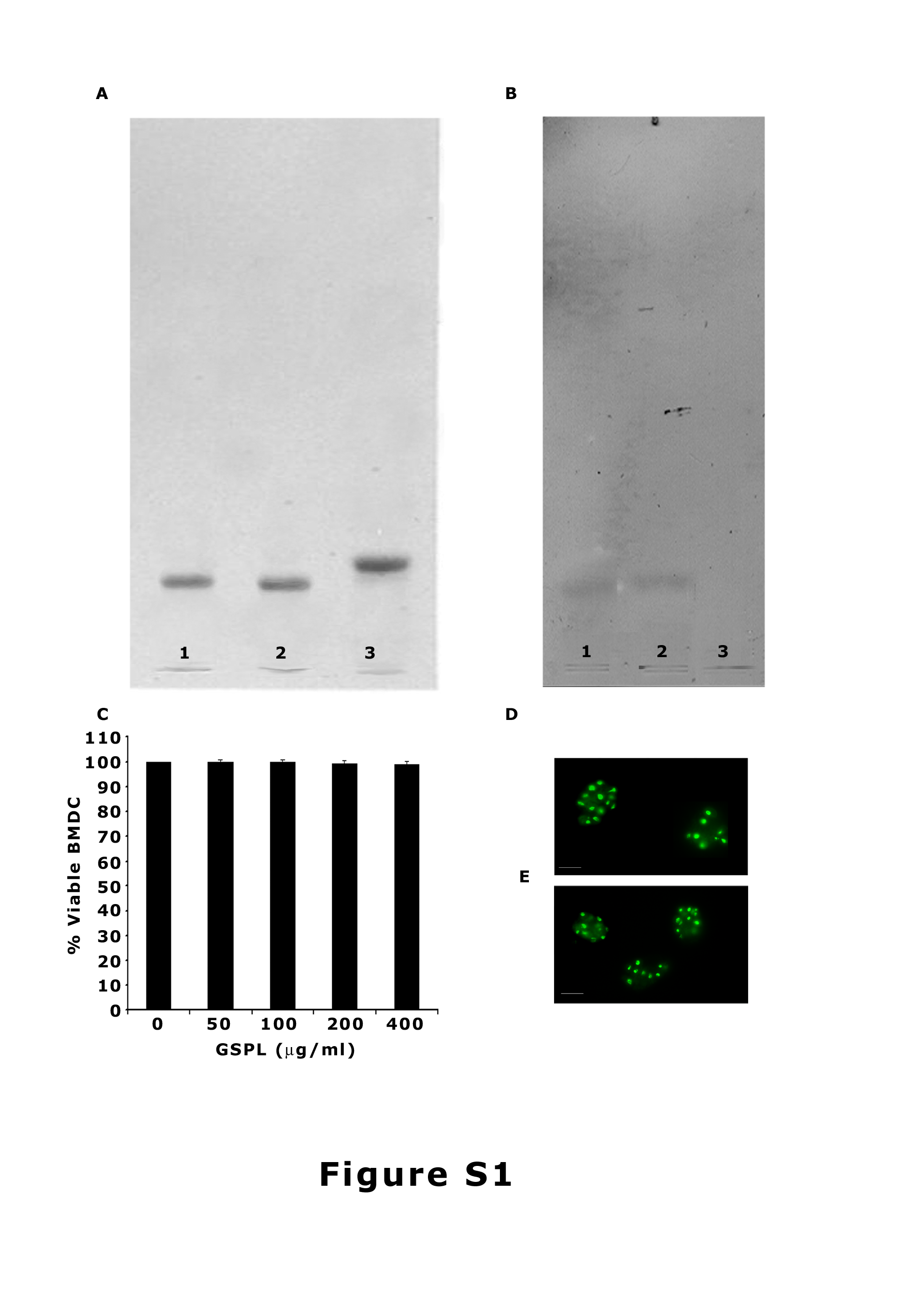

Supplement: Figure S1 — Thin layer chromatogram of GSPL and effect of GSPL on BMDC viability and phagocytic activity. (A,B) TLC of GSPL and β-glycosidase treated GSPL. Lane 1, GSPL; lanes 2 and 3, α-galactosidase and β-galactosidase treated GSPL respectively. The glycolipids in plate A were visualized with the diphenyl amine reagent for glycolipids. Plate B was transferred to PVDF membrane by TLC blotting and overlaid with biotinylated Erythrina cristagalli lectin. A representative chromatogram developed with chloroform∶methanol∶0.25N ammonia in 0.25% KCL(65∶45∶9) is shown. (C) BMDCs were incubated with various concentrations of GSPL (50–400 µg/mL) for 24 h. Cell viability was assessed by the MTT method. (D,E) Phagocytic activity of GSPL treated BMDCs. FITC-coupled latex beads were co-incubated with BMDCs in absence (D) and in presence of GSPL (E). Extracellular beads were removed by extensive washing; the cells were observed under fluorescence microscope. Scale bars, 10 µm. (TIF) [file ppat.1002646.s001.tif]

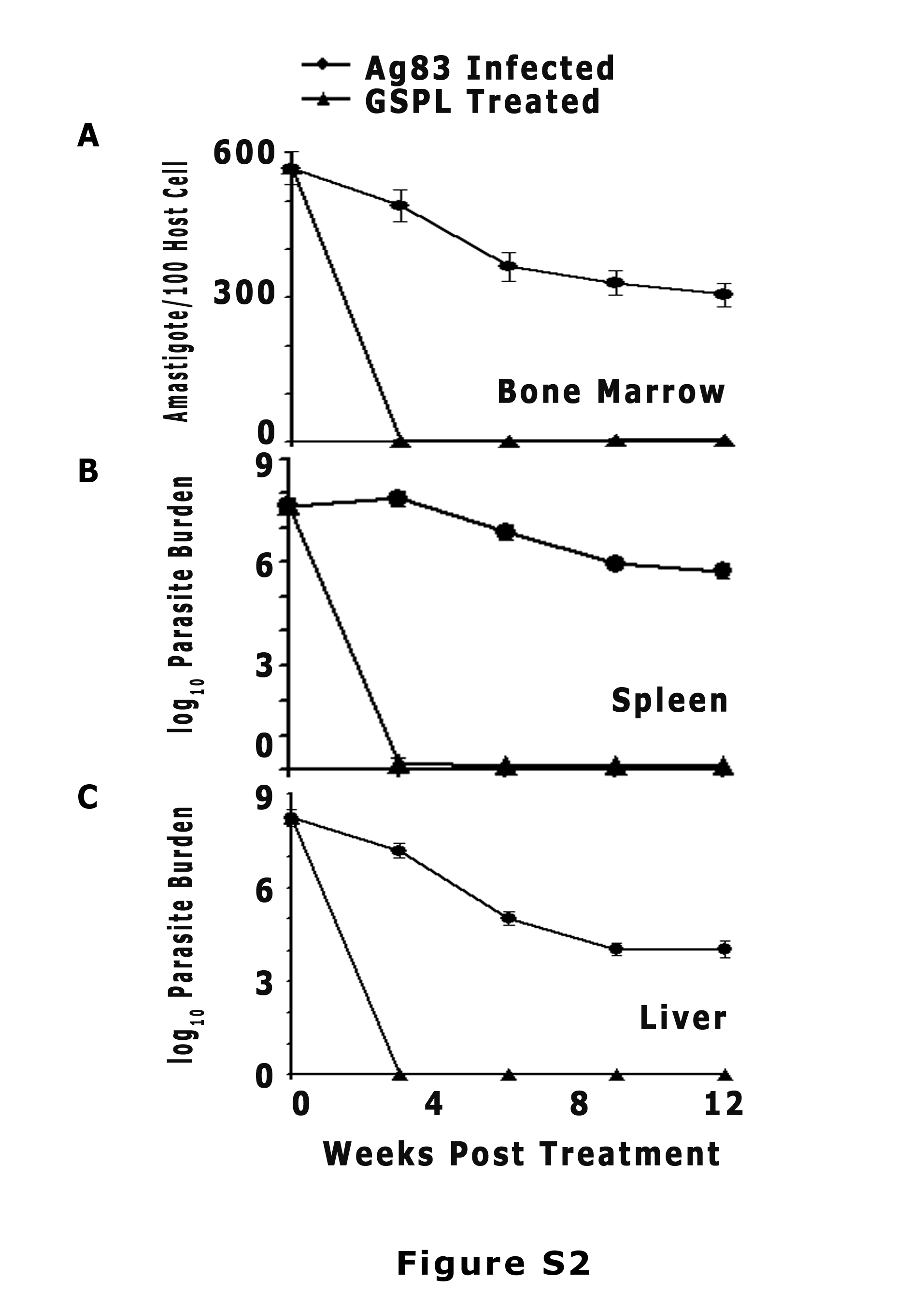

Supplement: Figure S2 — Protective effect of GSPL on disease relapse. LD-infected BALB/c mice were treated with GSPL (8 wk p.i.) and were sacrificed every 3 months up-to 12 months and compared to vehicle-treated infected controls. Bone marrow (A), splenic (B) and hepatic (C) parasite burdens were determined as described in Materials and Methods . Data represent the means ± SD of 5 animals per group and are representative of three individual experiments. p<0.0001 at all time points after infection, compared with respective infected control groups; paired two-tailed Student's t-test (TIF) [file ppat.1002646.s002.tif]

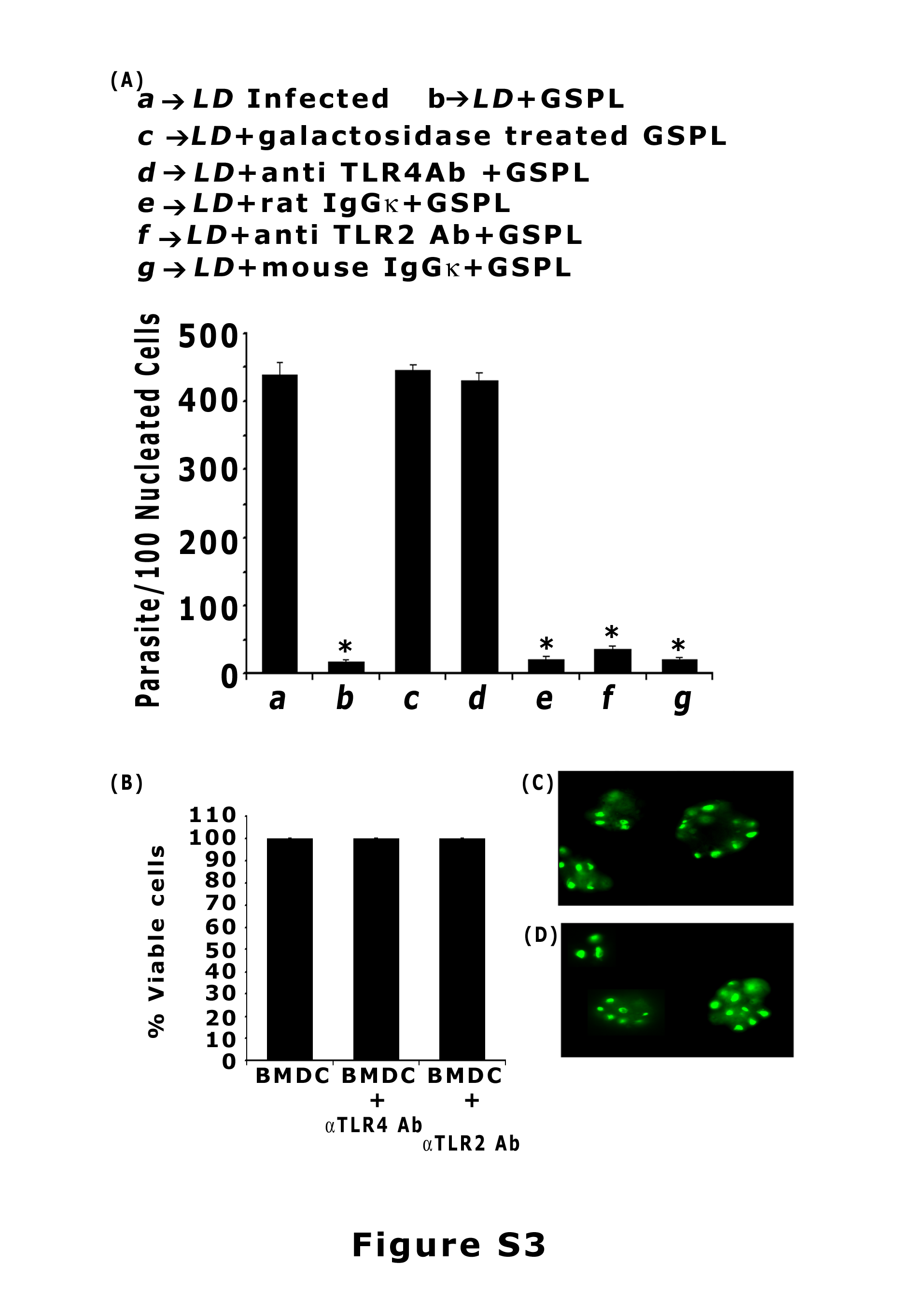

Supplement: Figure S3 — GSPL mediated protection depends on TLR4. (A) BMDCs were infected for 48 h with stationary phase 2nd passage LD promastigotes at a parasite/APC ratio of 20∶1. Infected BMDCs were treated with 100 µg/mL GSPL for 24 h. Anti TLR2 and anti TLR4 antibodies and isotype controls were added to parallel cultures and intracellular parasite number was determined by Giemsa staining. (B–D) The viability and phagocytic ability of the BMDCs treated with GSPL was assessed as described in legend to Figure S1. (B) Viability of GSPL treated BMDCs in presence of anti TLR2 and anti TLR4 antibodies. The phagocytic activity of BMDCs in absence (C) and presence (D) of GSPL. Experiments were done at least three times each and one set of representative data is shown. Error bars represent mean ± SD, n=3. * p<0.0001; paired two-tailed Student's t-test. (TIF) [file ppat.1002646.s003.tif]

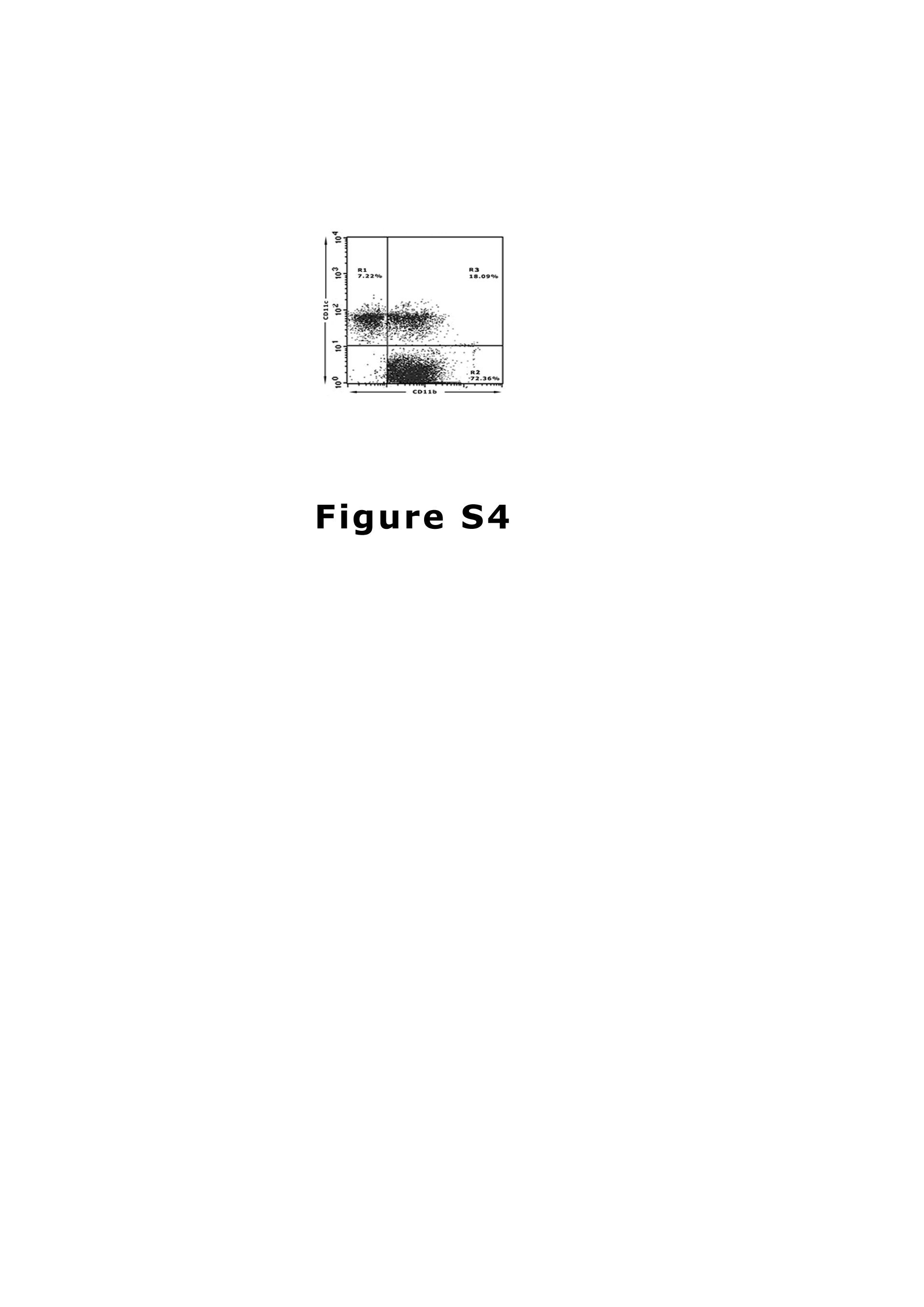

Supplement: Figure S4 — FACS analysis of splenic adherent cells. Shown is a representative APC sample. Region R1 defines all Cd11c+ cells; region R2 defines all Cd11b+ cells and region R3 defines all Cd11b+Cd11c+ cells. Experiment was done at least three times. (TIF) [file ppat.1002646.s004.tif]

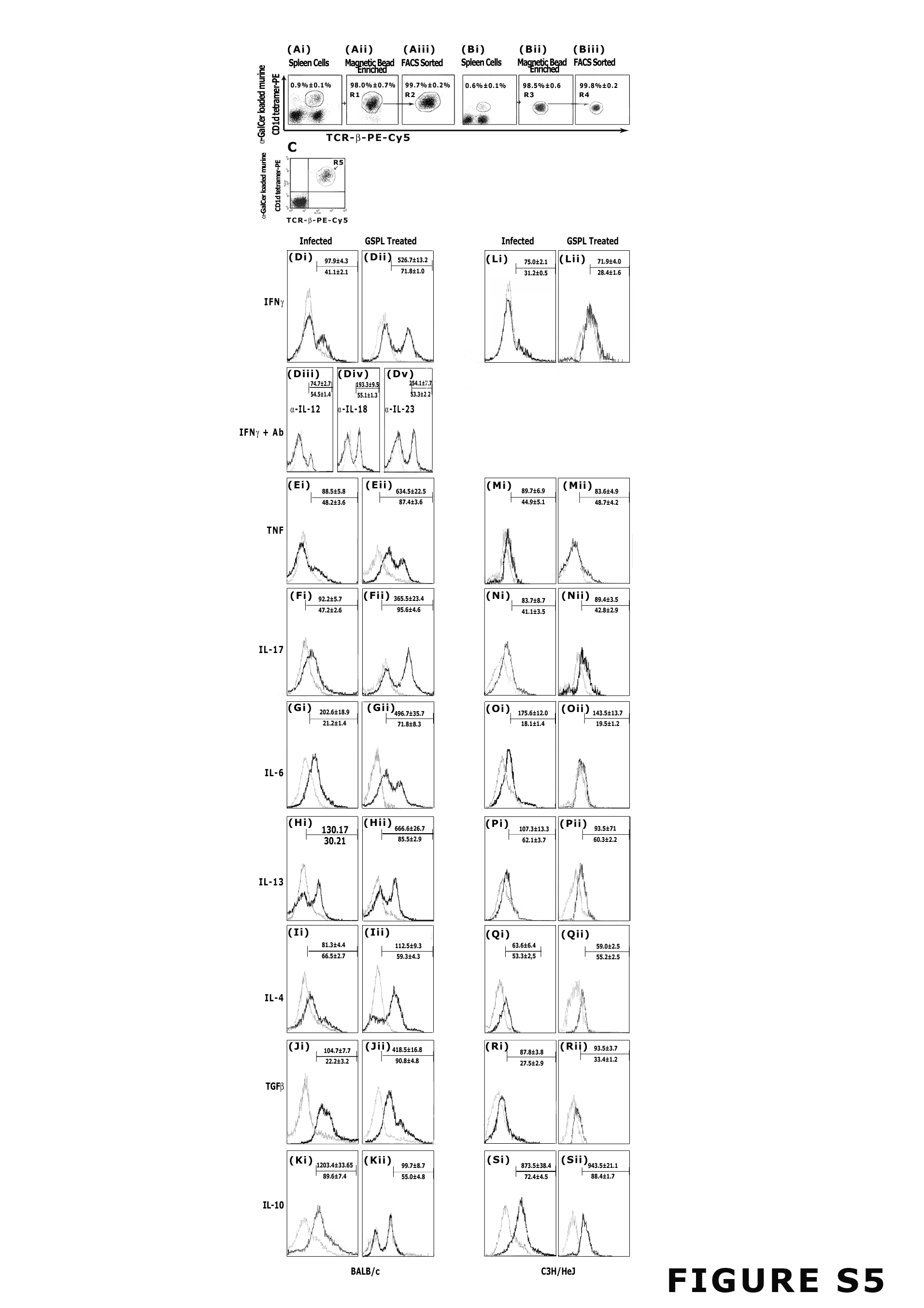

Supplement: Figure S5 — Intracellular flow cytometric analysis of cytokine profile in GSPL treated LD infected WT BALB/c and C3H/HeJ mice. Sixty days LD infected animals were treated with GSPL as described in the legend of Figure 1. NKT cells from spleens of individual experimental BALB/c (A) and C3H/HeJ (B) mice were identified as α-GC/CD1d tetramer+αβTCR+ cells (Ai,Bi) and after enrichment by magnetic cell sorting (Aii,Bii;R1,R3) were further purified by FACS sorting (Aiii,Biii;R2,R4). αGalCerCD1d-tetramer+TCRβ+iNKT cells isolated from experimental animals were mixed with autologous splenic adherent cells as described for Figure 4. Cells were either stimulated with GSPL (+GSPL) at 100 µg/mL or were treated with medium only (−GSPL) for the time periods as mentioned in the text. For the characterization of the cytokine profile, the T cells were then stained using appropriate concentrations of monoclonal antibody directed against the respective cytokines. To specifically identify iNKT cells, we gated on cells that doubly stained with fluorescent PE–CD1d–α-GalCer tetramers and PE-Cy5-anti-TCRβ (C;R5). The gated cells were further analyzed for the expression of intracellular FITC-labeled cytokines. Numbers above horizontal bars represent mean fluorescence intensity and numbers below the bar represents percentage of cytokine positive cells. Anti IL-12p70, anti IL-18 and anti IL-23p19 Abs or isotype controls (data not shown) were added to parallel cultures and intracellular IFN-γ production was determined by FACS (Diii–v)). D,L, IFN-γ; E,M, TNF-α; F,N, IL-17A; G,O, IL-6; H,P, IL-13; I, Q, IL-4; J,R, TGF-β and K, S, IL-10 expressions. Gray lines depict un-stimulated controls and black lines indicate GSPL stimulated cell. Data represent the mean ± SD for five animals per group. Data are representative of three experiments. (TIF) [file ppat.1002646.s005.tif]

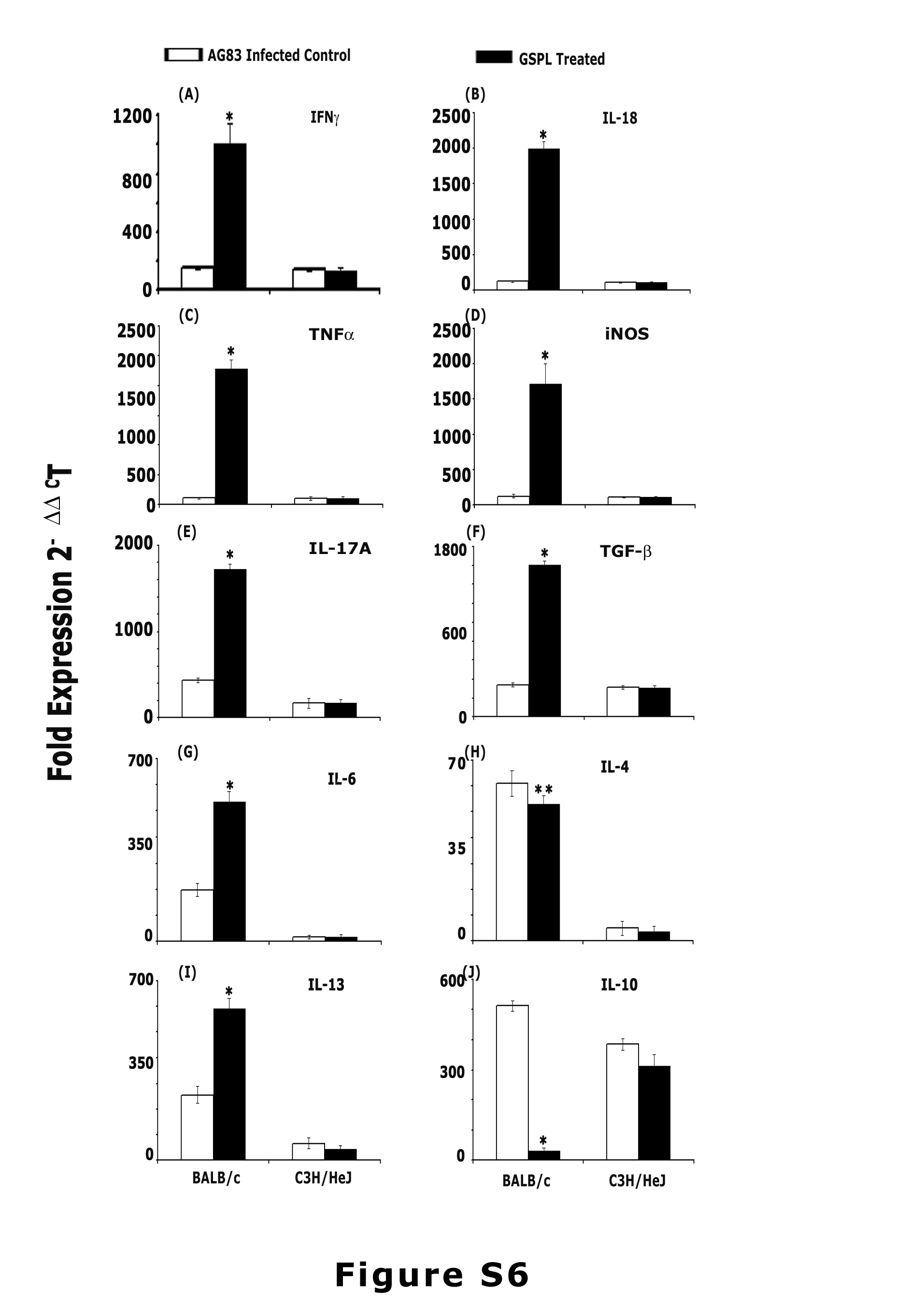

Supplement: Figure S6 — GSPL mediated induction of Th1/Th17 cytokines mRNA. Gene expression was done by comparative CT method using real-time PCR. Fold change in m-RNA expression profiles of A, IFN-γ; B, IL-18; C, TNF-α; D, iNOS; E, IL-17A; F, TGF-β; G, IL-6; H, IL-4; I, IL-13 and J, IL-10 in splenic lymphocytes of LD-infected (infected control) and LD-infected-GSPL treated (GSPL treated) mice. Results show mean ± SD of five individual mice per group (*p<0.0001; **p<0.05 versus corresponding infected control; paired two-tailed Student's t-test). Each gene was normalized to the housekeeping gene (β-actin) before fold change was calculated to account for variations between different samples. (TIF) [file ppat.1002646.s006.tif]

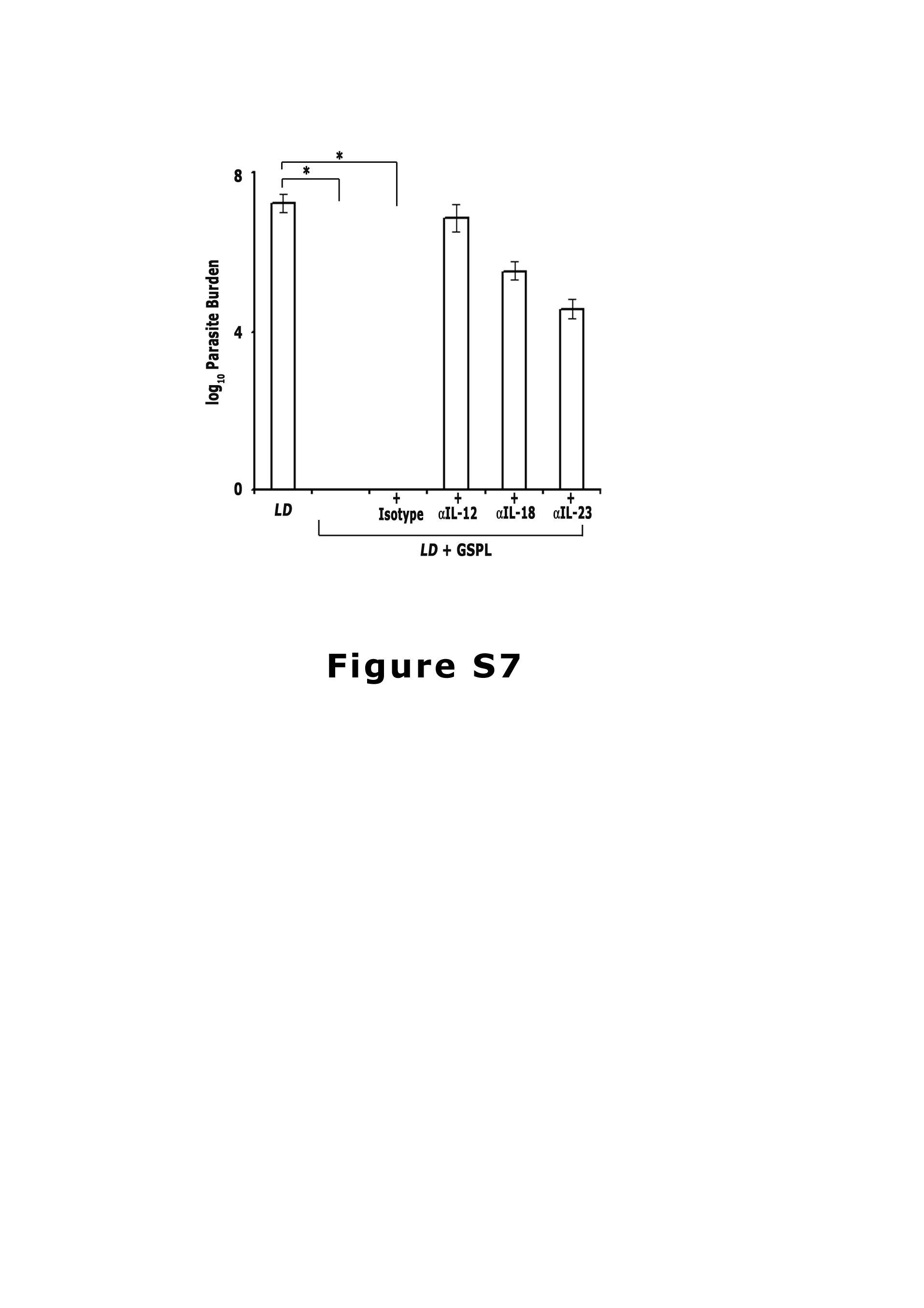

Supplement: Figure S7 — Anti-IL-12p70, anti IL-18, and anti IL-23p19 treatment of LD infected BALB/c mice reduced the anti-leishmanial effect of GSPL. Sixty days LD infected BALB/c mice were injected i.p. on days −1, 0, and +1 of GSPL treatment either with isotype control antibody, anti-IL-12p70, anti IL-18, or IL-23p19. Splenic parasite burden was determined as described in Figure 1. Data represent the mean ± SD of 3 animals per group, and are representative of three individual experiments. *p<0.0001 compared with the LD infected control groups; paired two-tailed Student's t-test. (TIF) [file ppat.1002646.s007.tif]

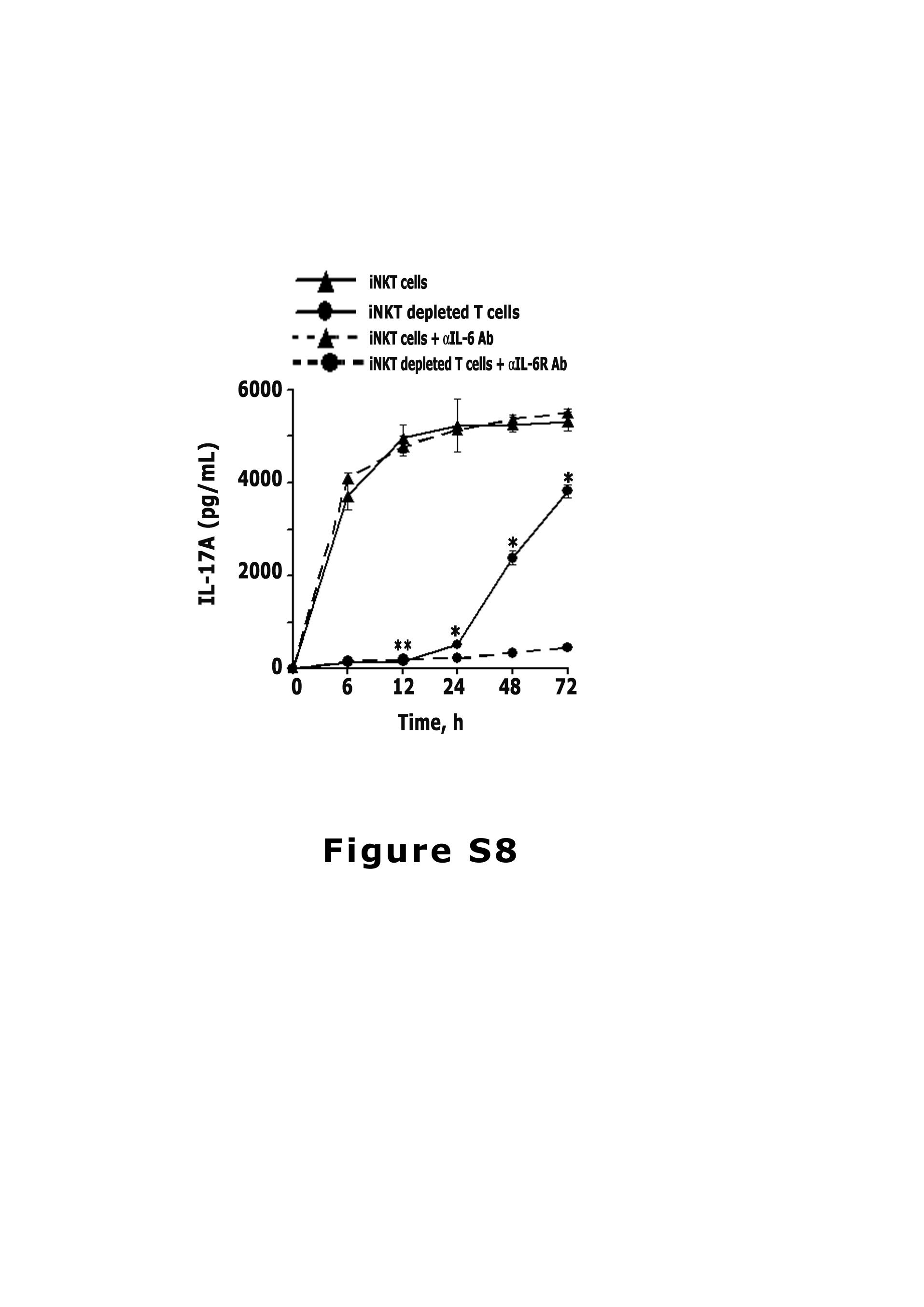

Supplement: Figure S8 — Kinetics of IL-17A production by iNKT cells of cured mice. Sixty days LD infected animals were treated with GSPL as described in the legend of Figure 1. Animals were sacrificed 15 days after the last treatment. iNKT cells and NKT depleted cell populations from spleens of individual experimental BALB/c mice were isolated as described in legend to Figure 6. Isolated T-cells were co-cultured with 1∶10 of autologous splenic adherent cells as described for Figure 4. Cells were stimulated with GSPL at 100 µg/mL, ± IL-6 Ab or ± IL-6R Ab for the time periods indicated and IL-17A in spleen cell culture supernatants were determined by ELISA. Results show mean ± SD of three individual mice per group; *p<0.0001 versus corresponding Ab treated group; paired two-tailed Student's t-test. Results of one from three independent experiments are shown. (TIF) [file ppat.1002646.s008.tif]

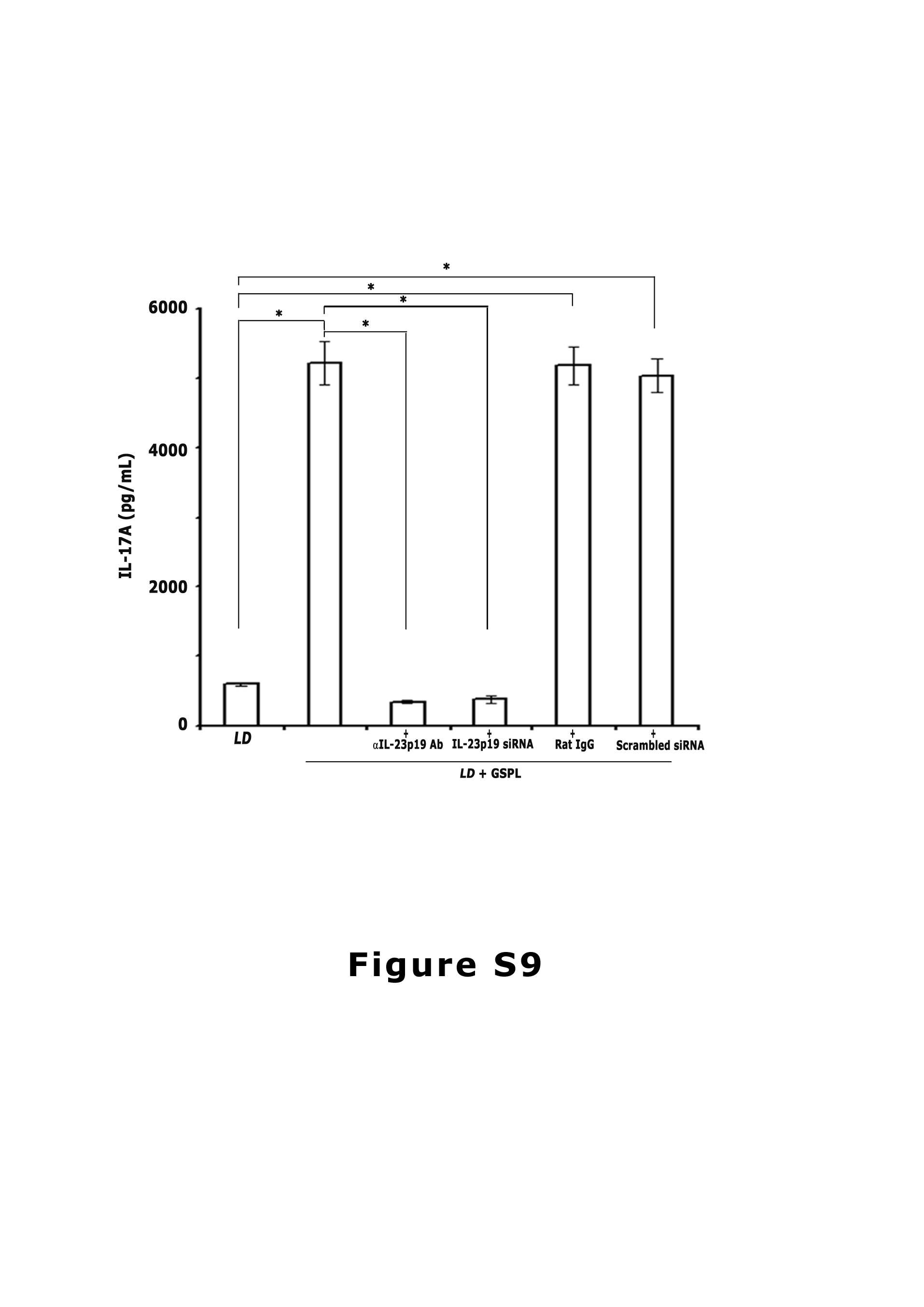

Supplement: Figure S9 — GSPL induces IL-17, which is dependent on IL-23. LD infected BALB/c mice were treated with GSPL or with vehicle control only. After 15 days, adherent spleen cells co-cultured with autologus iNKT cells were stimulated with GSPL as described in Figure 6, with or without anti IL-23 Ab or isotype controls. IL-17 was estimated in the supernatants by ELISA. IL-23 siRNA transfected adherent splenic cells were similarly cultured in parallel. Data represent the mean ± SD of 3 animals per group, and are representative of three individual experiments. *p<0.0001; paired two-tailed Student's t-test. (TIF) [file ppat.1002646.s009.tif]
