# Supplementary material for: TLR4 and NKT Cell Synergy in Immunotherapy against Visceral Leishmaniasis
Source: PLoS Pathog. 2012 Apr 12;8(4):e1002646. doi: 10.1371/journal.ppat.1002646 (PMC3325212; doi:10.1371/journal.ppat.1002646)
Supplement: Table S1 — Real-time RT-PCR primer sequences. (DOC) [file ppat.1002646.s010.doc]

Table S1: Real-time RT-PCR primer sequences.

| Protein/gene | Forward Primer(5’-3’) | ReversePrimer(5’-3’) |
| --- | --- | --- |
| IL-23p19 | cagcagctctctcggaat | acaaccatcttcacactggatacg |
| IL-12p35 | acagcaccagcttcttcatcag | tcttcaaaggcttcatctgcaa |
| IL-6 | acacatgttctctgggaaatcgt | aagtgcatcatcgttgttcataca |
| IL-17 | accgcaatgaagaccctgat | tccctccgcattgacaca |
| TLR2 | gccaccatttccacggact | ggcttcctcttggcctgg |
| TLR4 | agaaattcctgcagtgggtca | tctctacaggtgttgcacatgtca |
| GAPDH | ggtgaaggtcggtgtgaacgga | gagggatctcgctcctggaaga |
